# Supplementary material for: Chemodiversity and Biotechnological Potential of Microginins
Source: Int J Mol Sci. 2025 Jun 25;26(13):6117. doi: 10.3390/ijms26136117 (PMC12250027; doi:10.3390/ijms26136117)
Supplement: Supplementary file 1 [file ijms-26-06117-s001.zip › Figure S1 - Microginin Structures.pdf]

Figure S1. Chemical structures of microginin peptides in cyanobacteria.

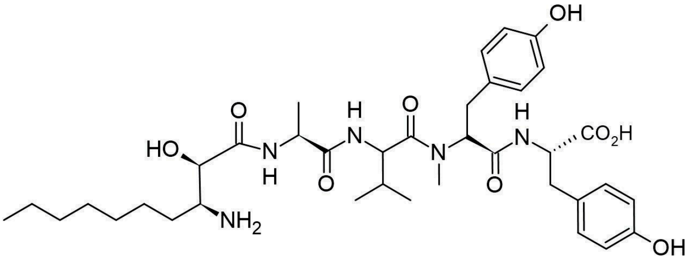

Microginin 1

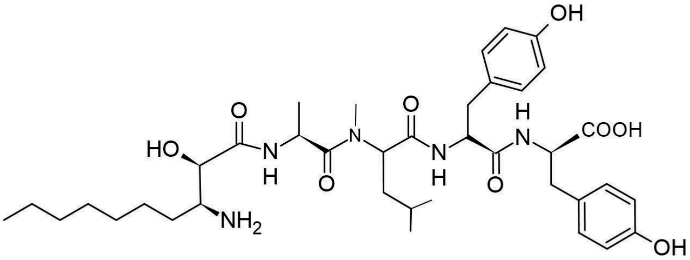

Microginin FR1

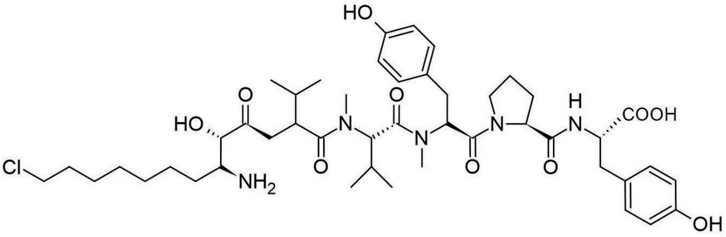

Microginin 299-A

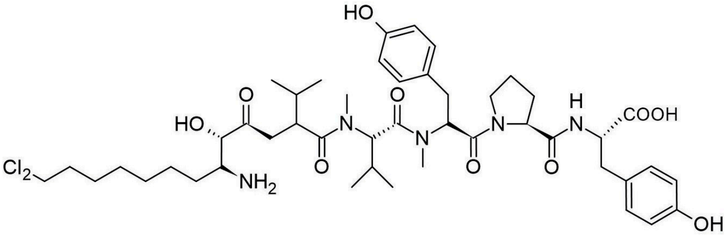

Microginin 299-B

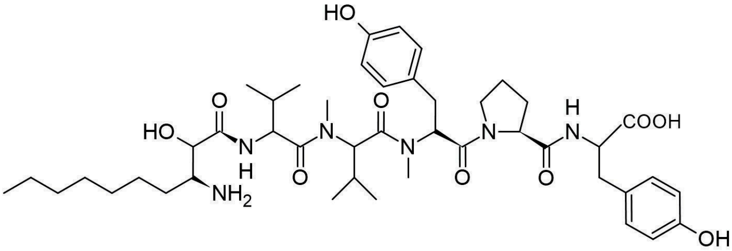

Microginin 299-C

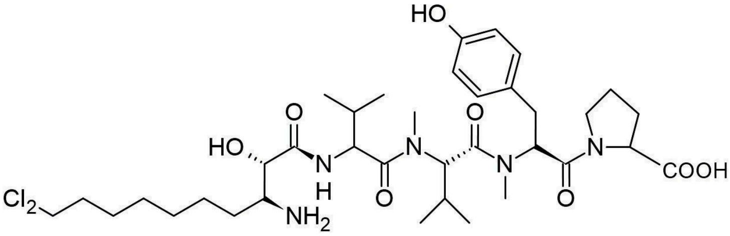

Microginin 299-D

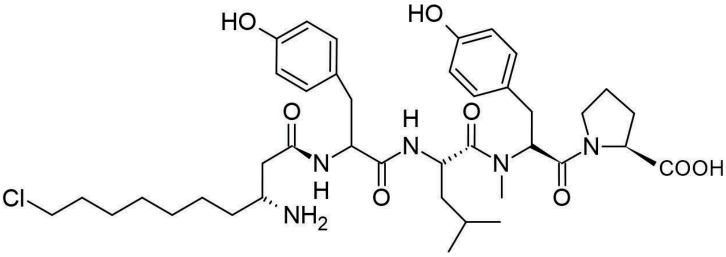

Microginin 99-A

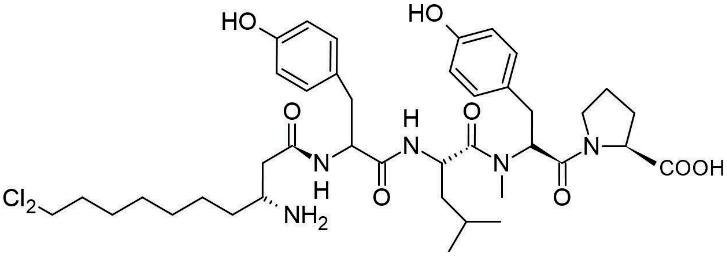

Microginin 99-B

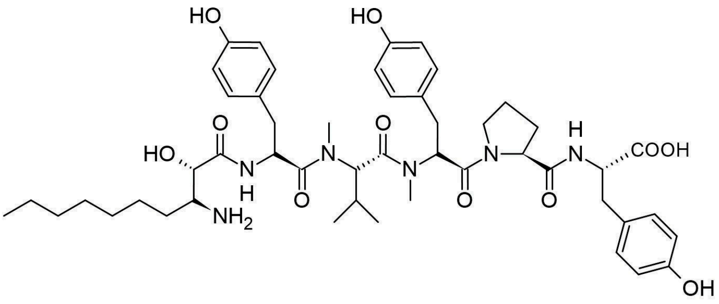

Microginin 51-A

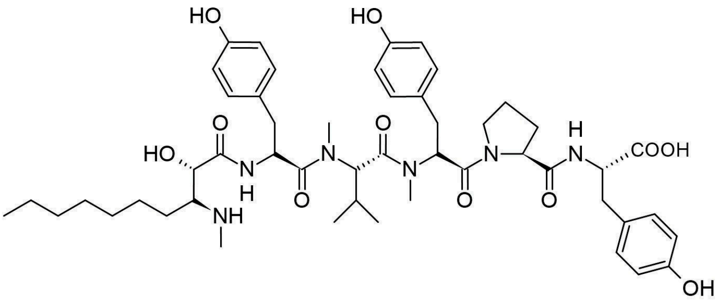

Microginin 51-B

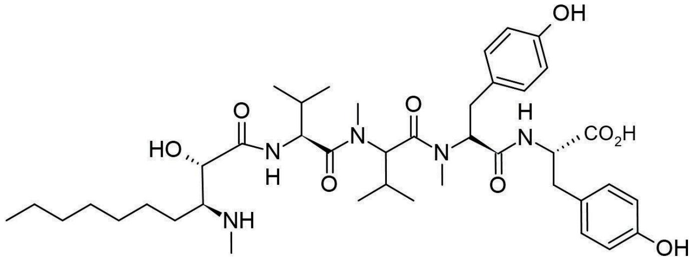

Microginin 478

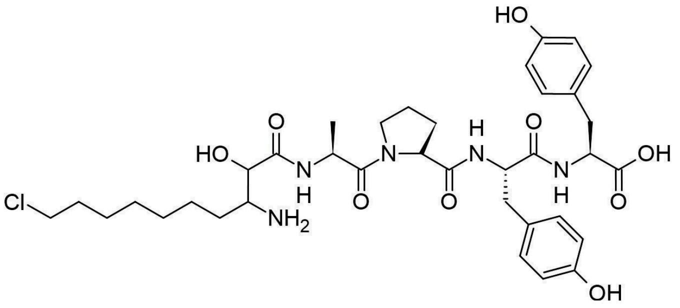

Microginin T1

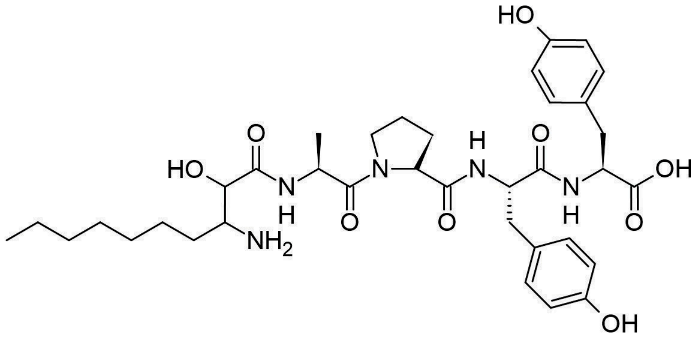

Microginin T2

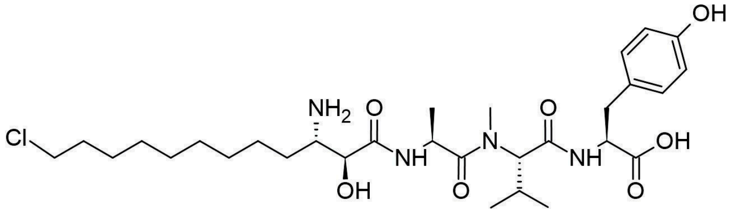

Microginin AL584

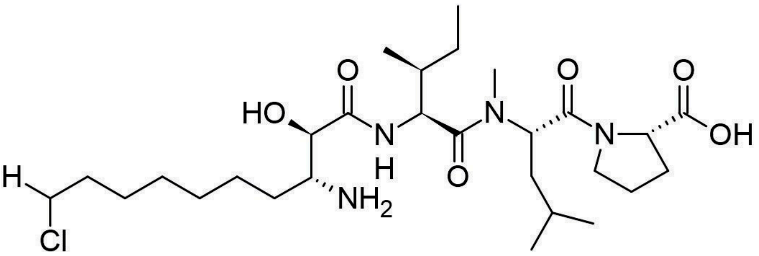

Microginin 91-A

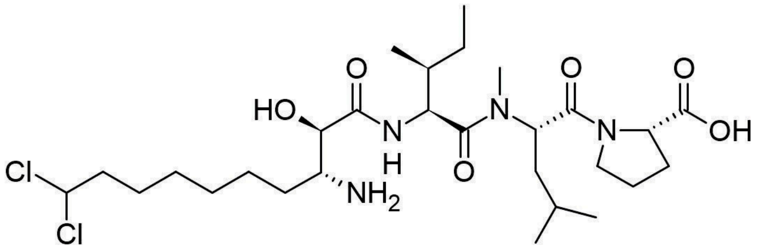

Microginin 91-B

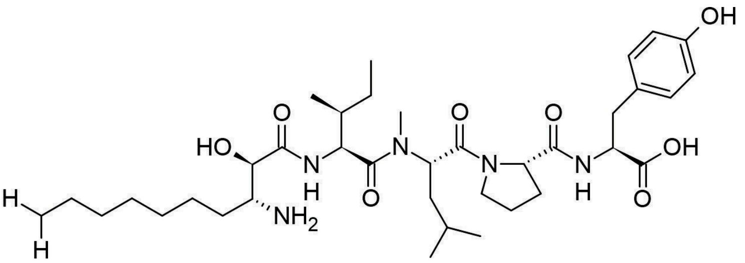

Microginin 91-C

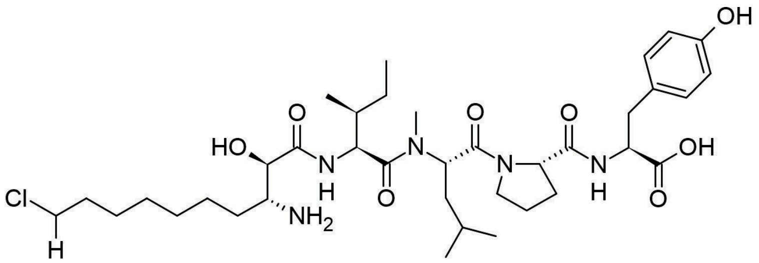

Microginin 91-D

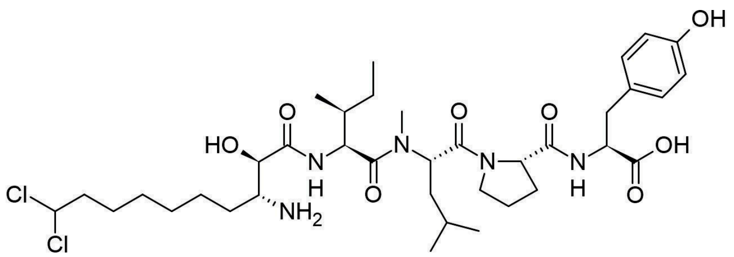

Microginin 91-E

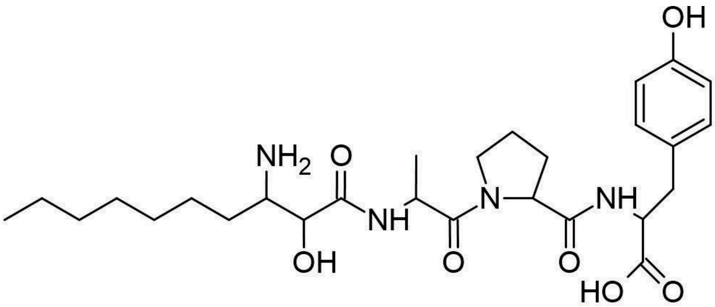

Microginin 535

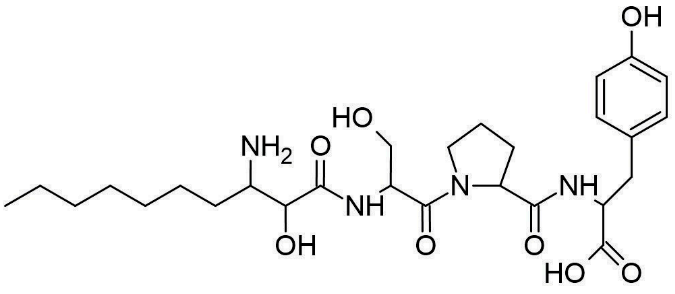

Microginin 551

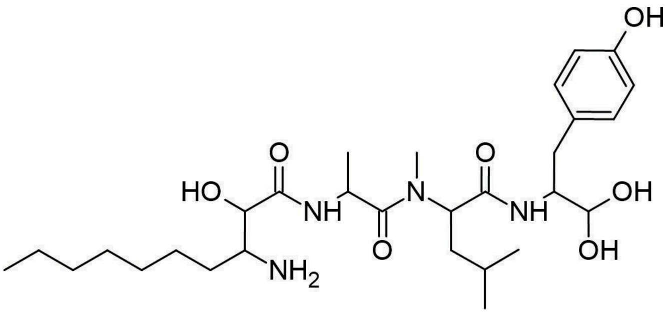

Microginin 565A

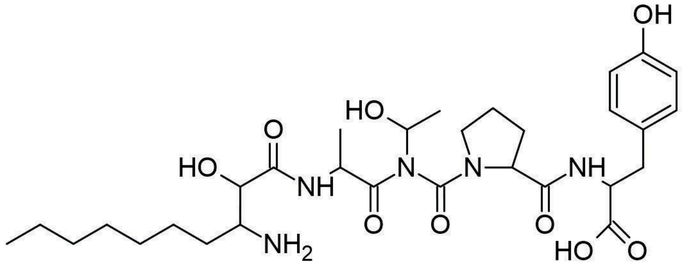

Microginin 565B

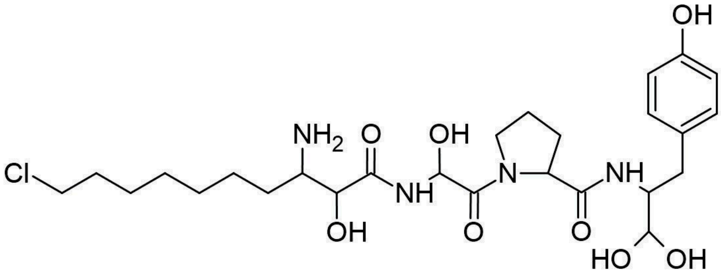

Microginin 568

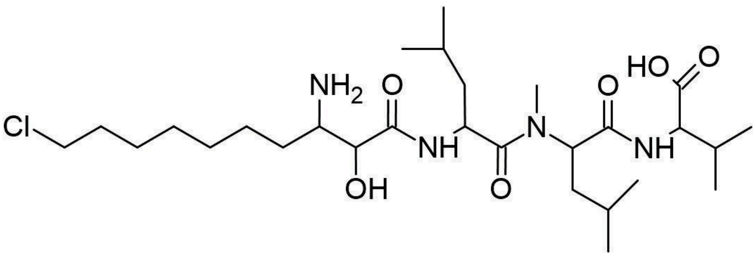

Microginin 576

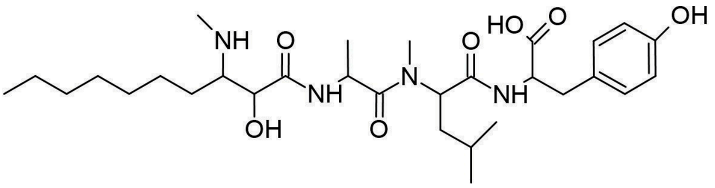

Microginin 579A

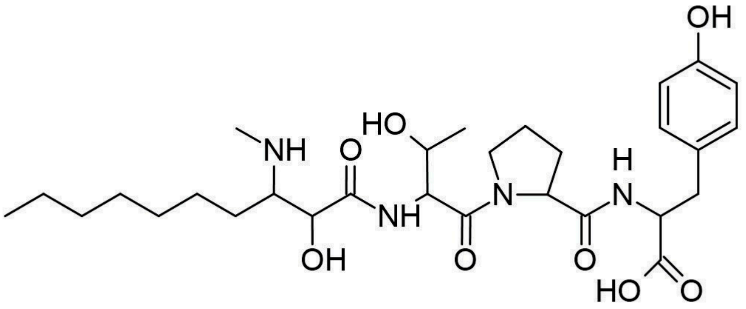

Microginin 579B

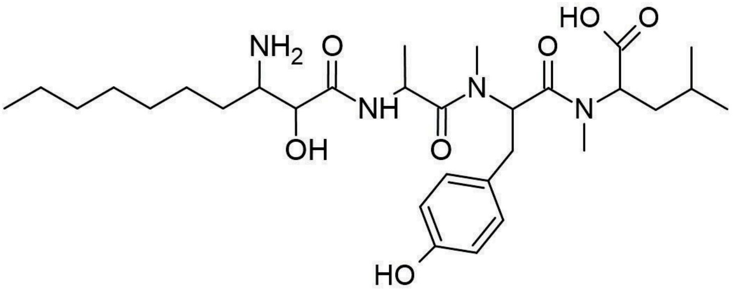

Microginin 579C

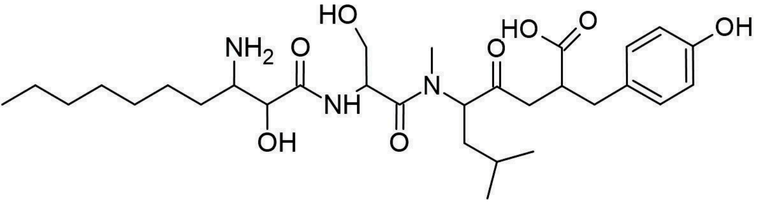

Microginin 581

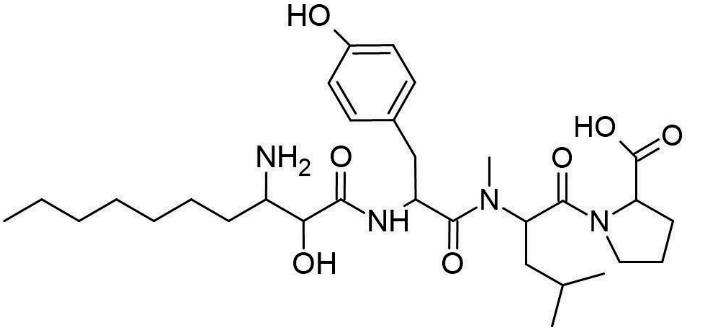

Microginin 591B

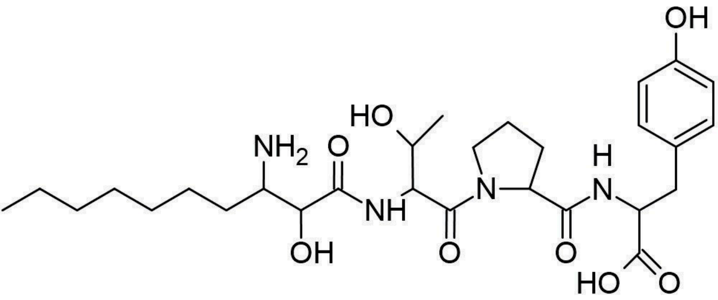

Microginin 598

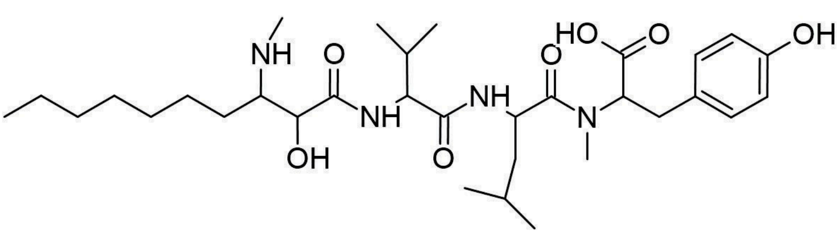

Microginin 607A

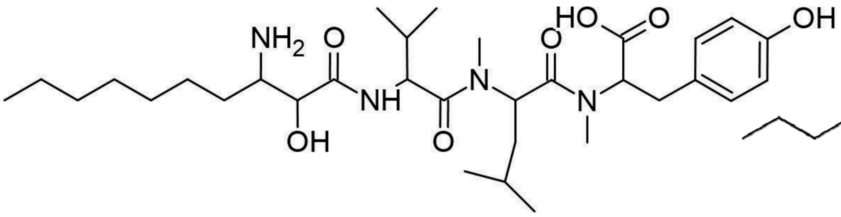

Microginin 607B

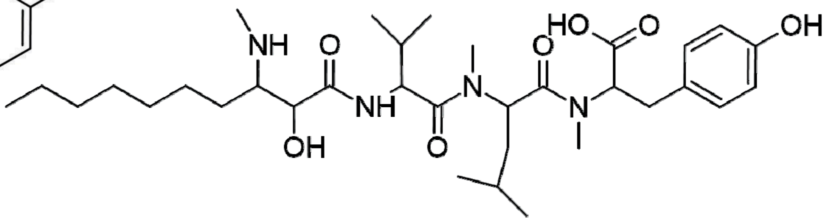

Microginin 621A

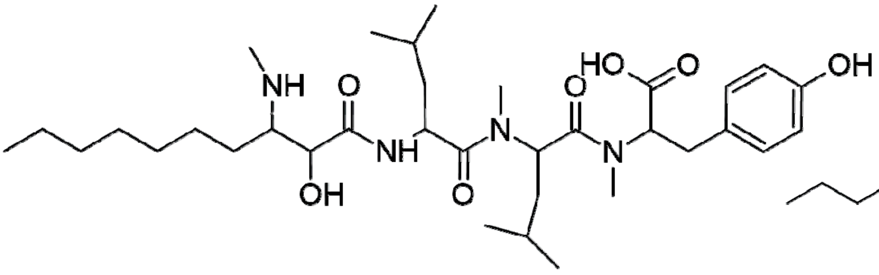

Microginin 621B

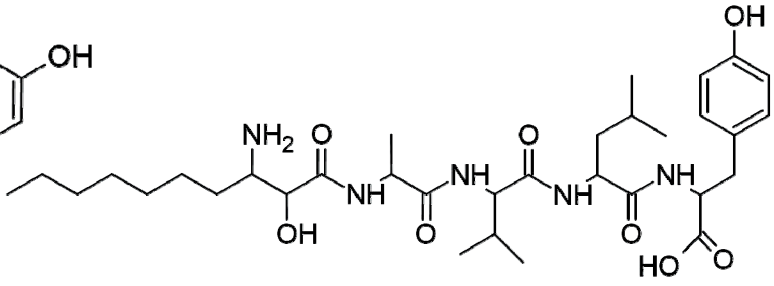

Microginin 650

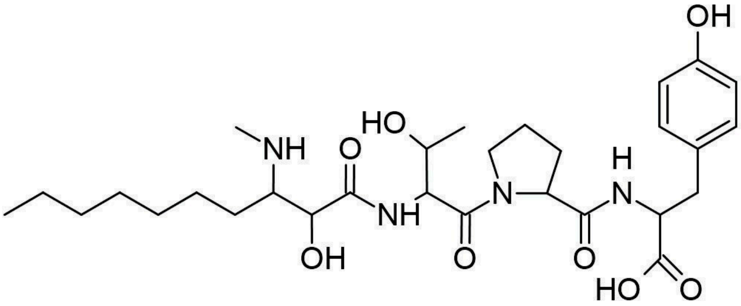

Microginin 683

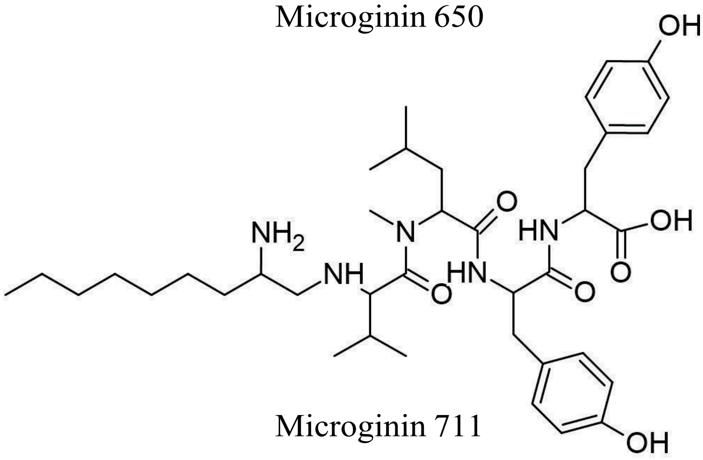

Microginin 711

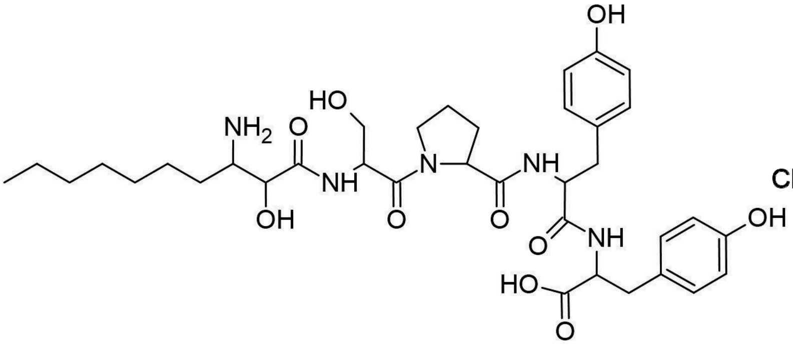

Microginin 714B

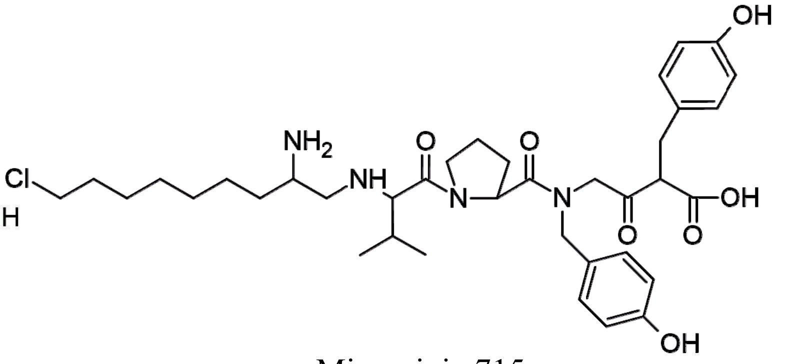

Microginin 715

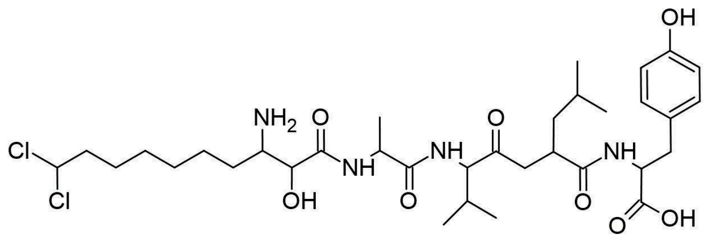

Microginin 717

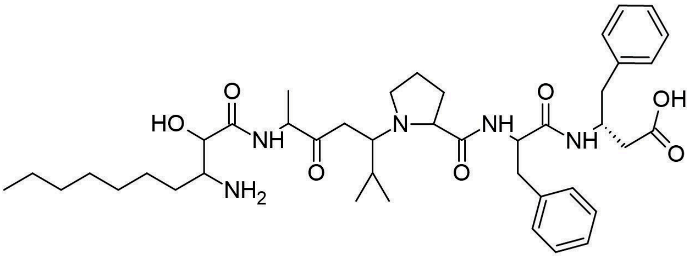

Microginin FR5

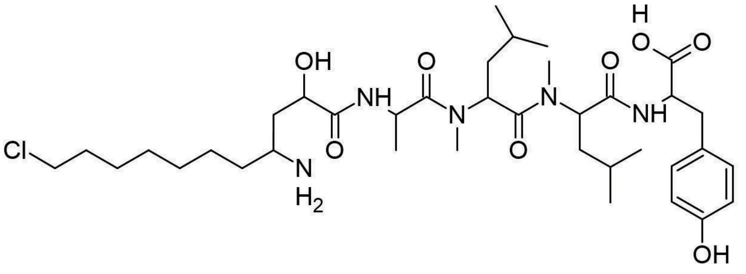

Microginin 725

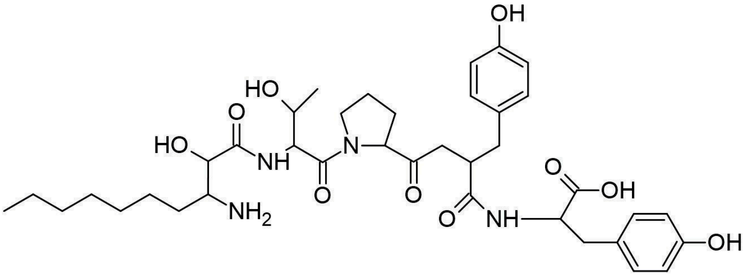

Microginin FR3

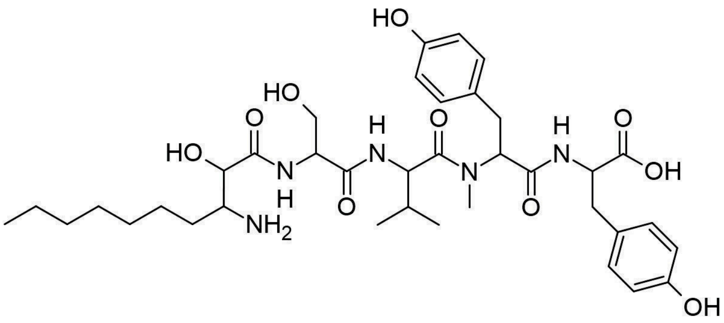

Microginin 730

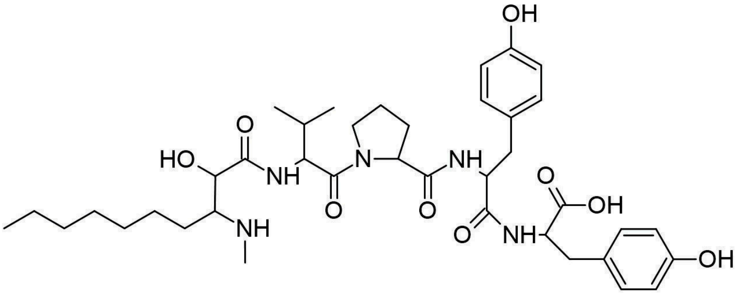

Microginin FR6

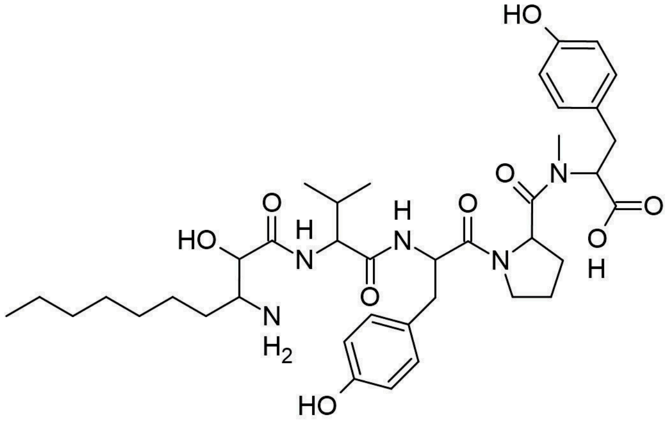

Microginin 740B

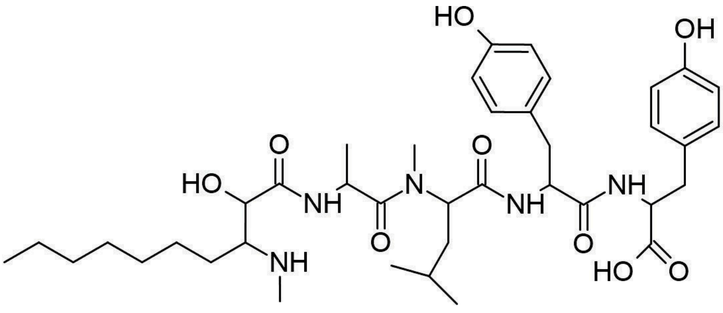

Microginin 742A

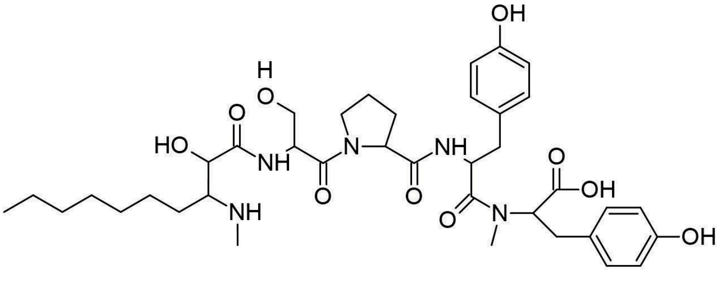

Microginin 742B

Microginin 742C

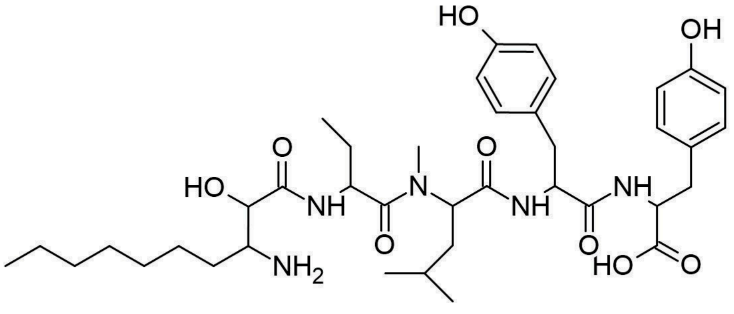

Microginin 744

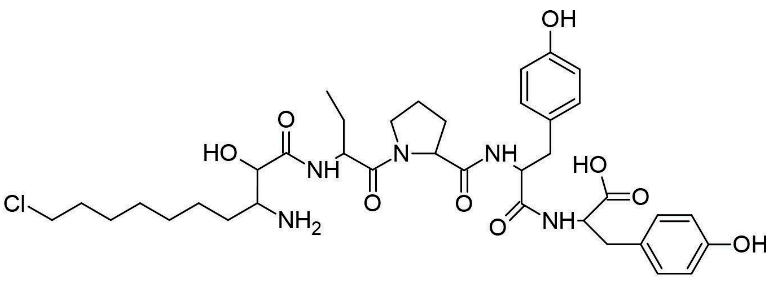

Microginin 747A

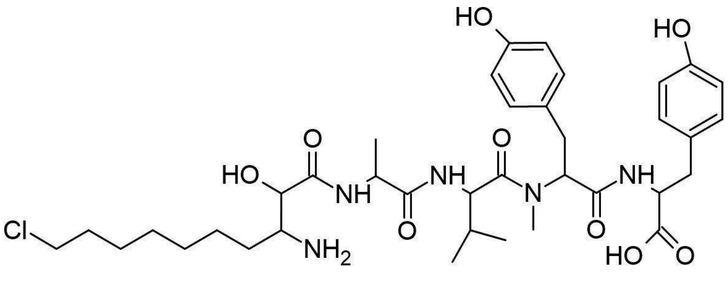

Microginin 747B

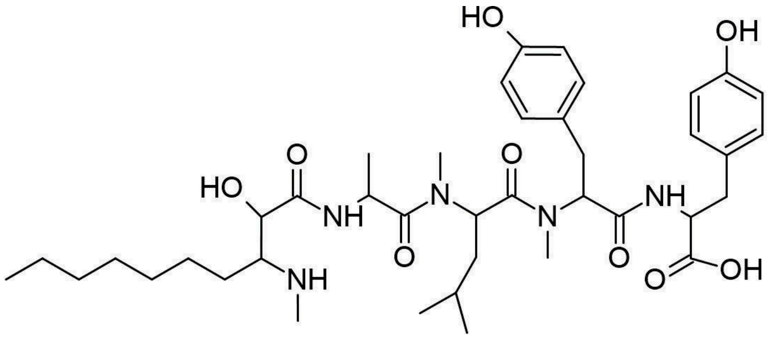

Microginin 756B

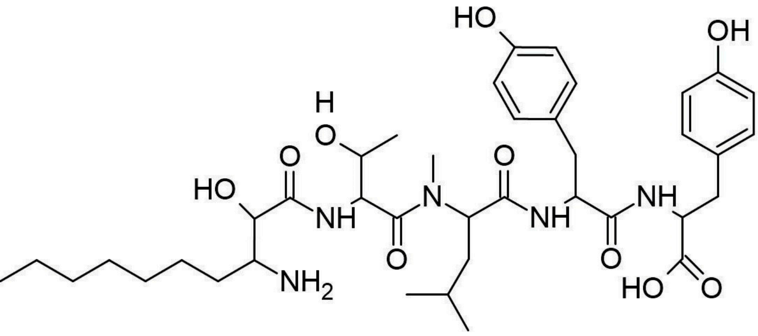

Microginin 757

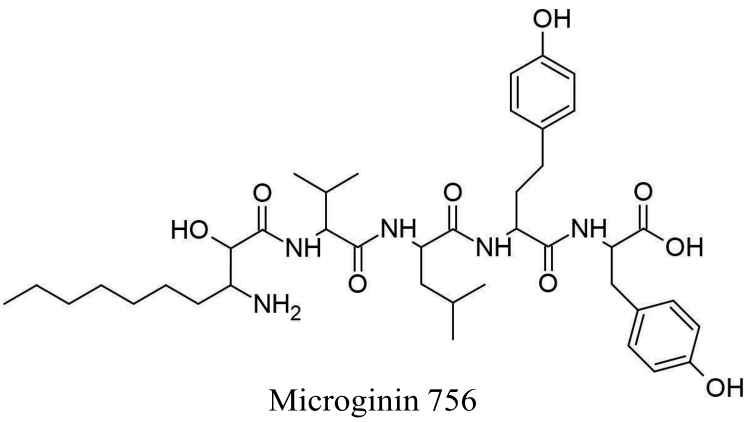

Microginin 756

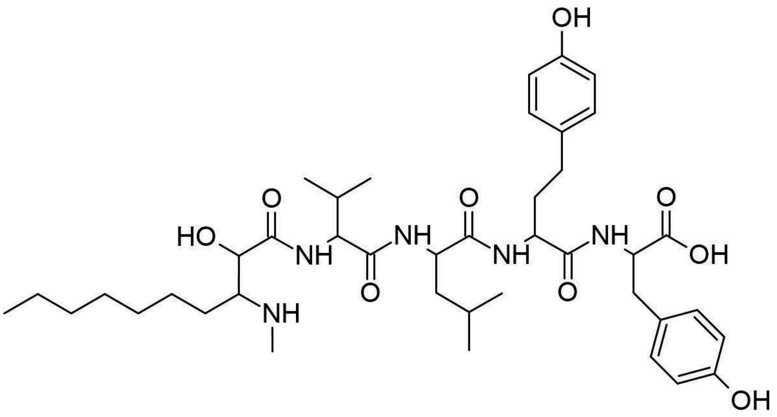

Microginin 770

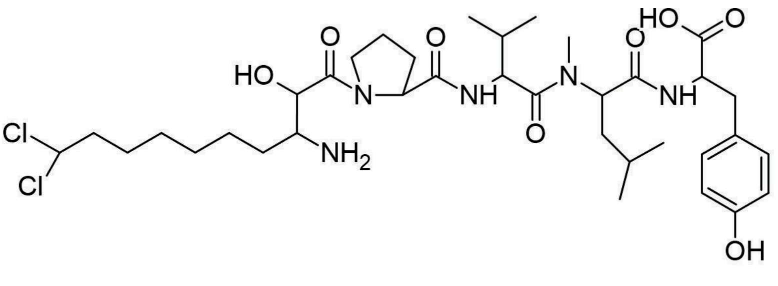

Microginin 757B

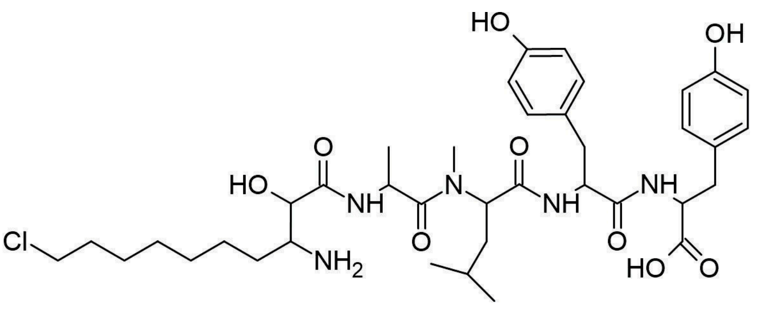

Microginin 761A

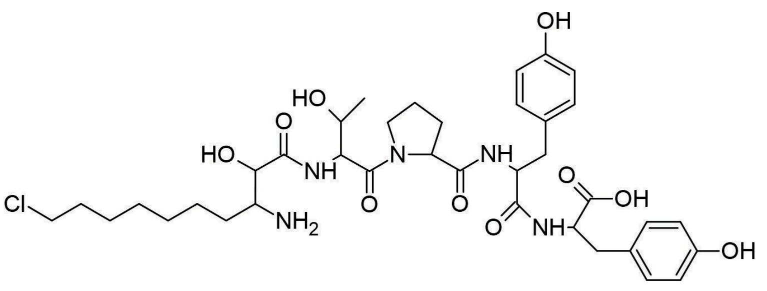

Microginin 761B

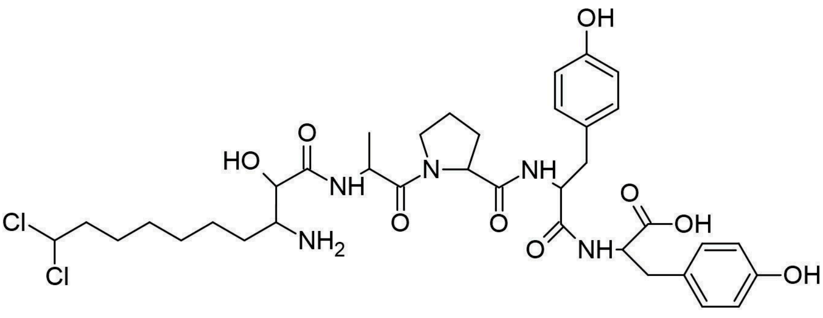

Microginin 765

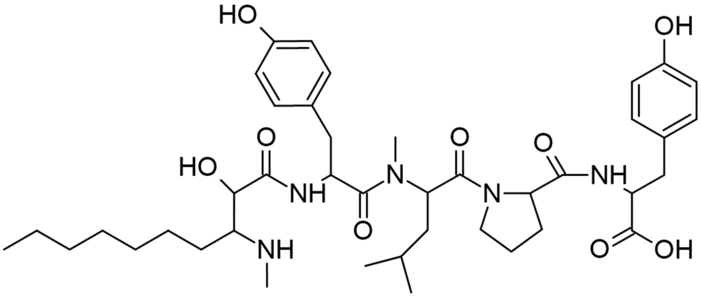

Microginin 767

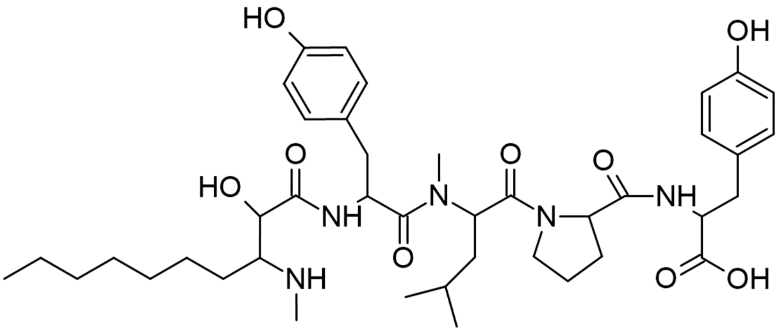

Microginin KR767

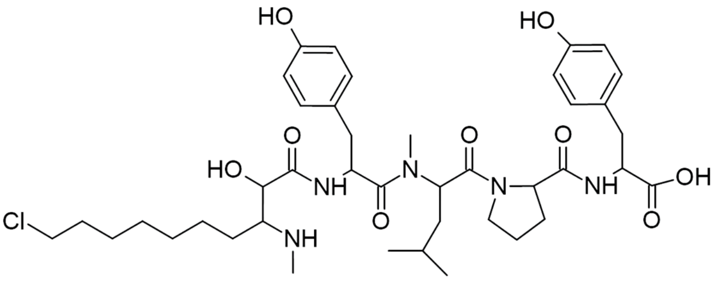

Microginin KR801

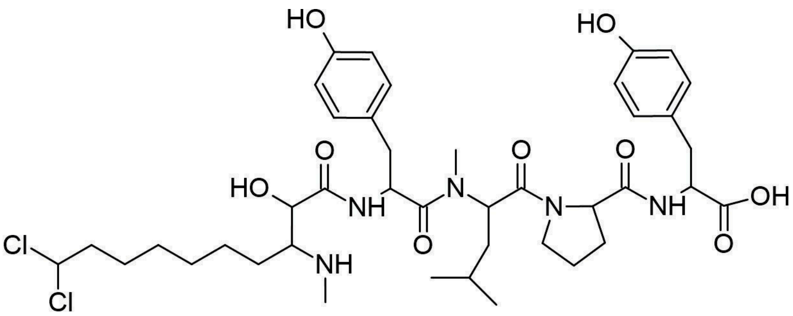

Microginin KR835

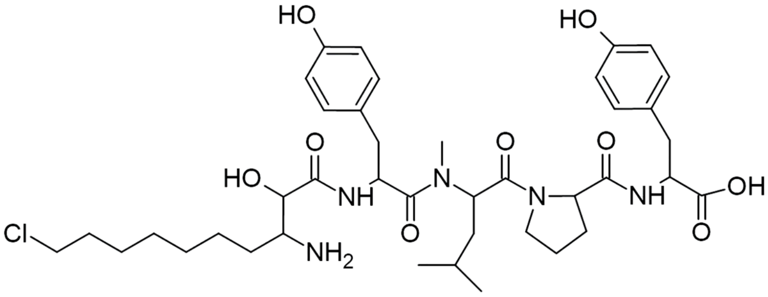

Microginin KR787

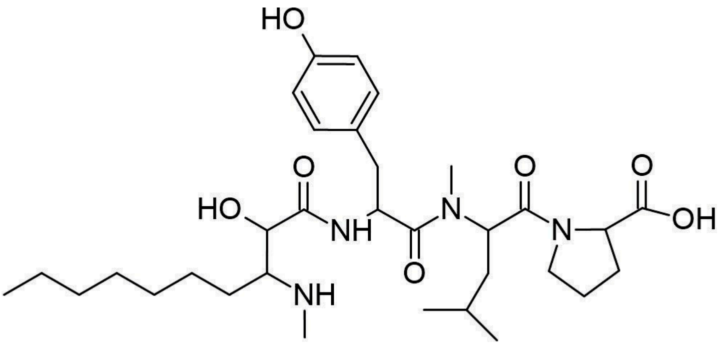

Microginin KR604

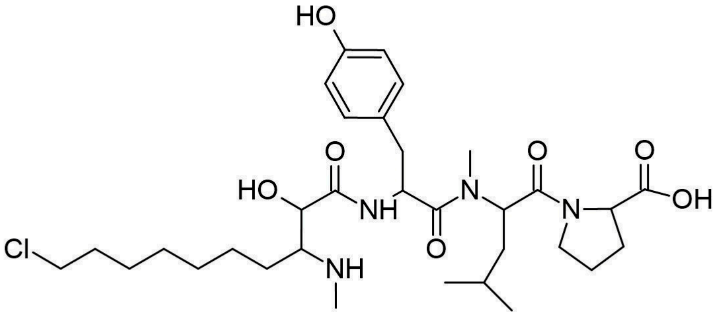

Microginin KR638

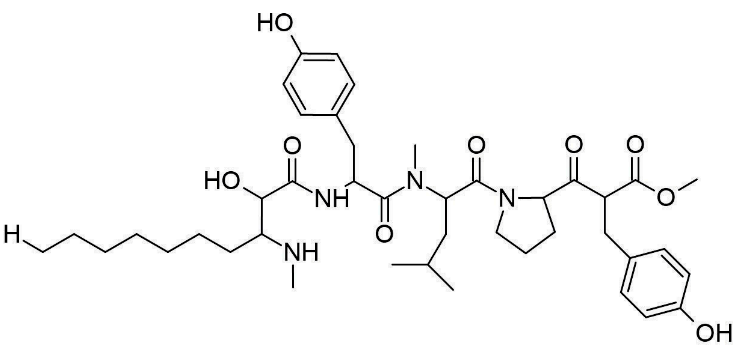

Microginin KR781

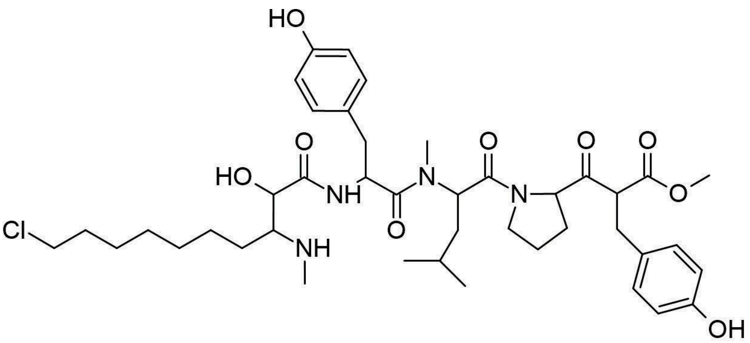

Microginin KR815

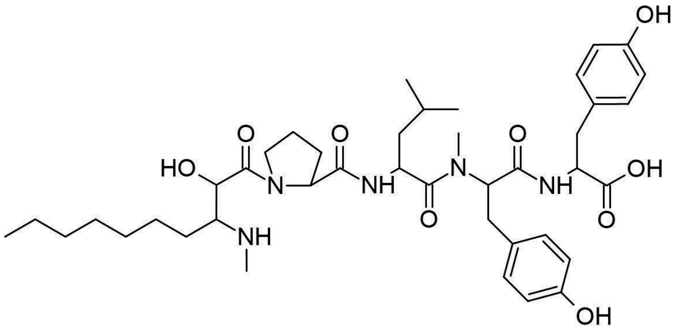

Microginin FR8

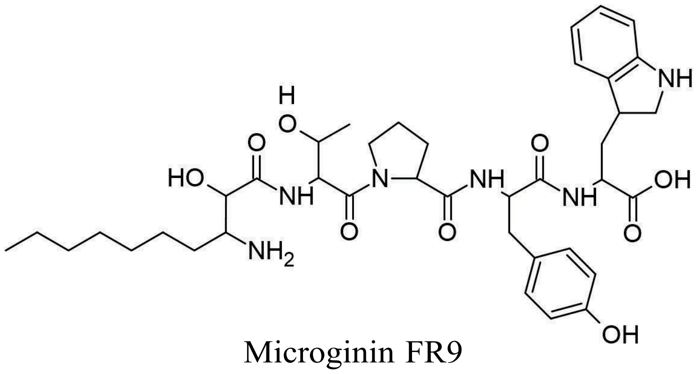

Microginin FR9

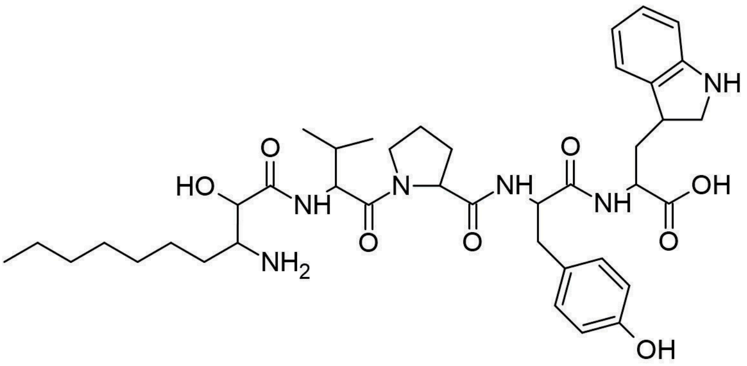

Microginin FR10

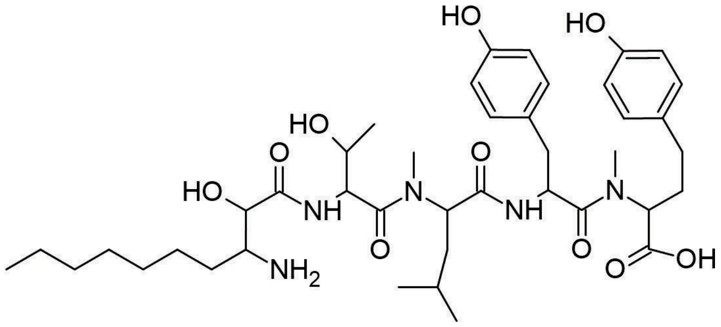

Microginin 772

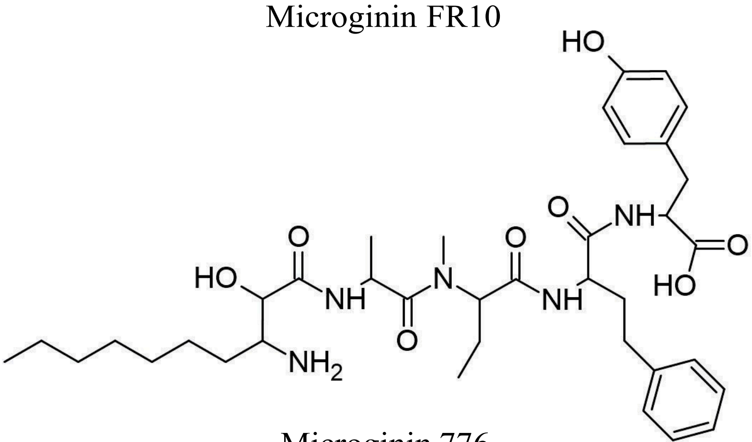

Microginin 776

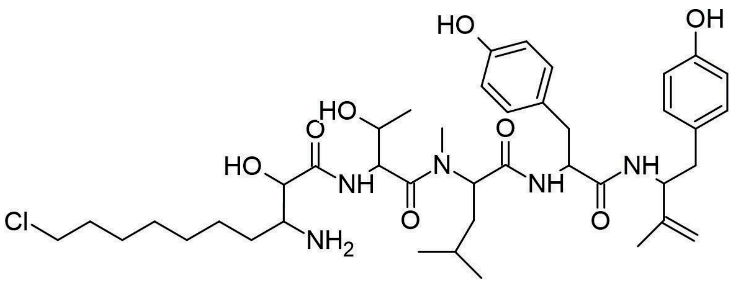

Microginin 791

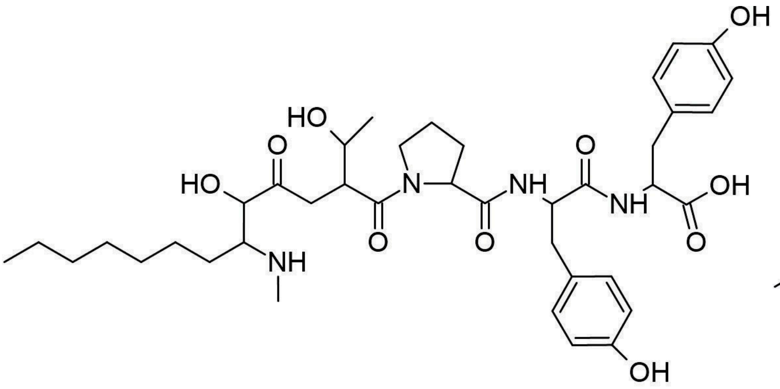

Microginin FR2

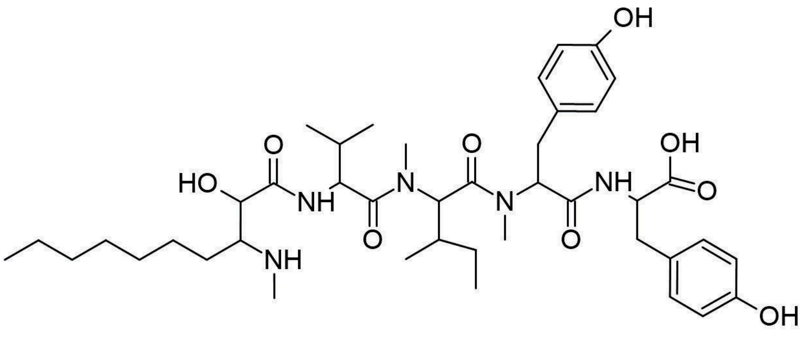

Microginin SD-755

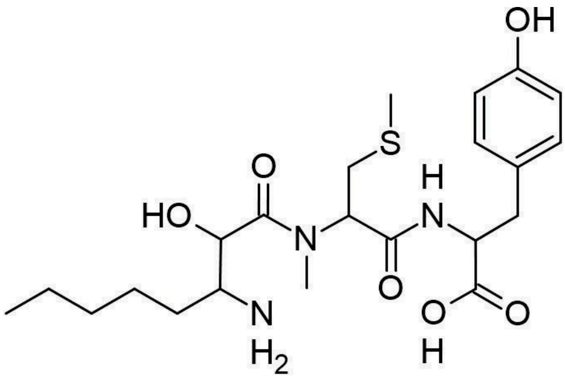

Microginin 511

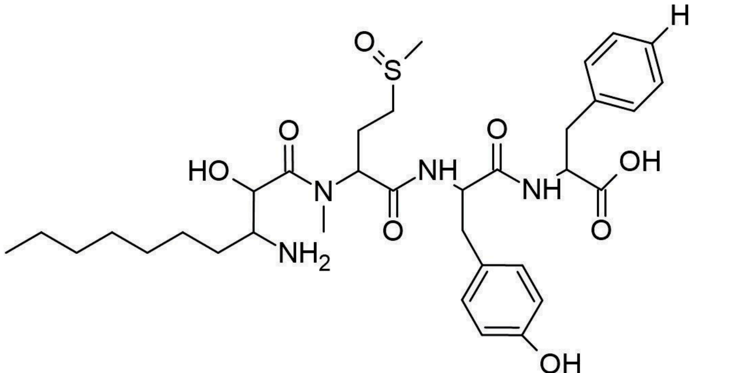

Microginin 690

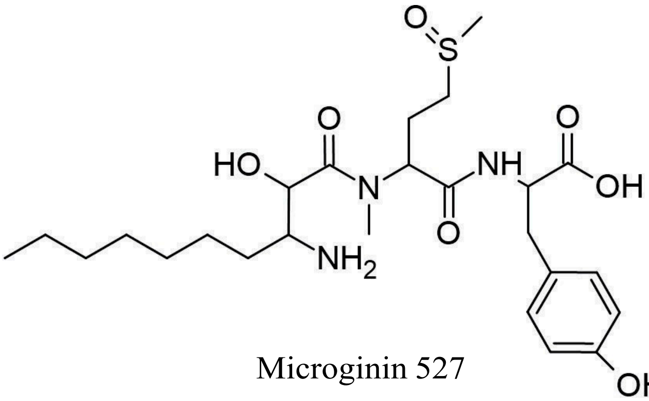

Microginin 527

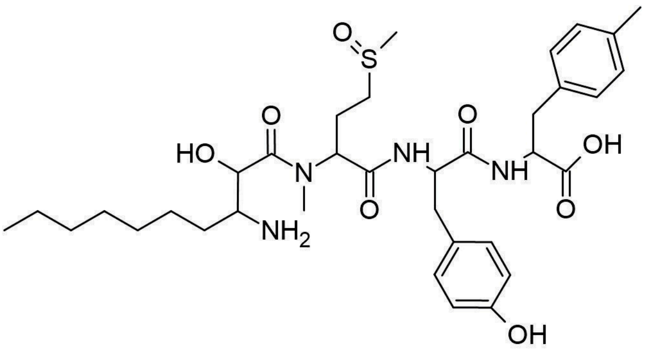

Microginin 704

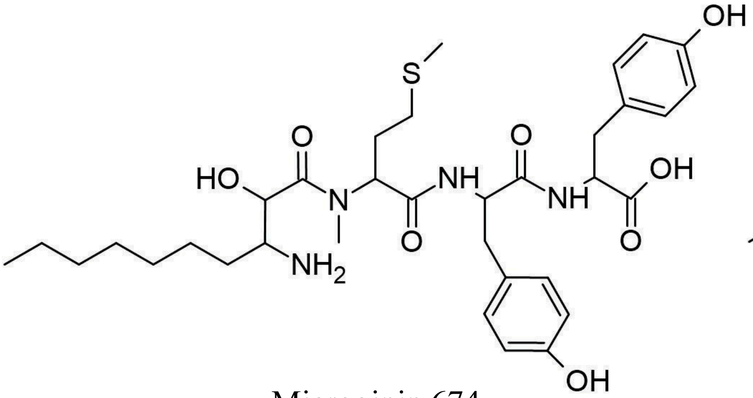

Microginin 674

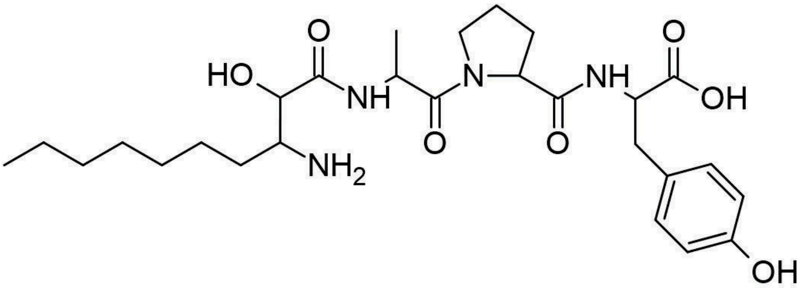

Microginin 549

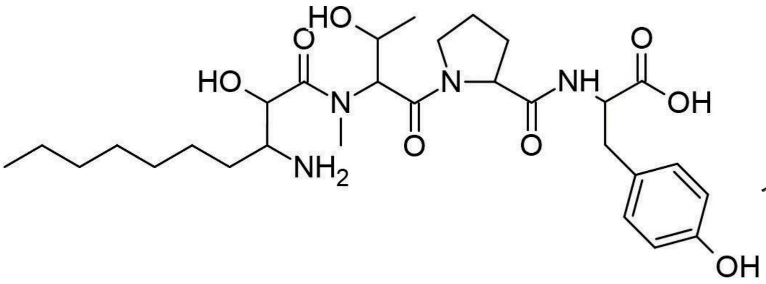

Microginin 595

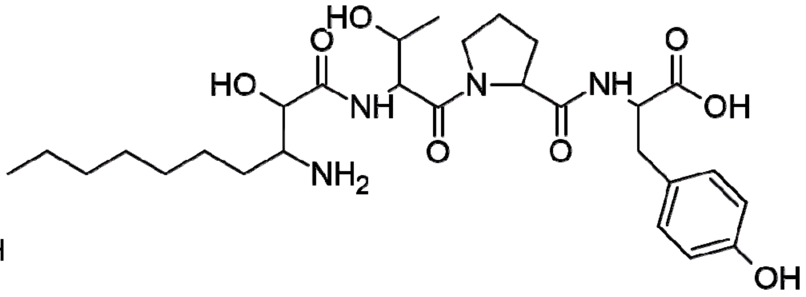

Microginin 581B

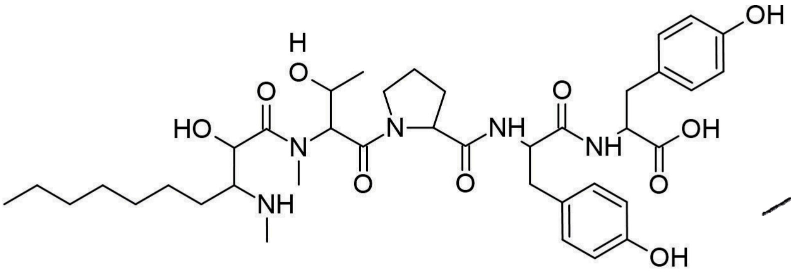

Microginin FR4

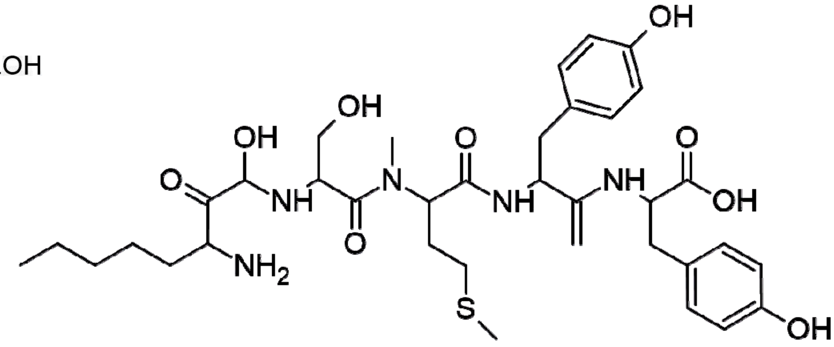

Microginin FR7

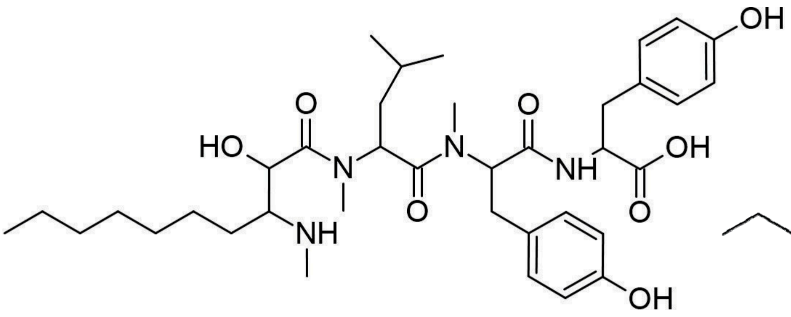

Microginin FR12

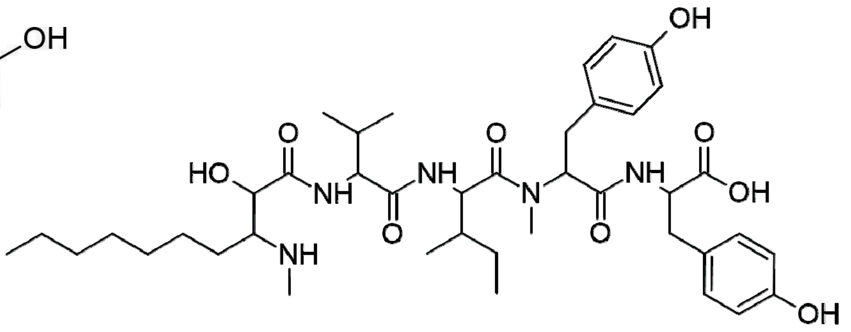

Microginin FR13

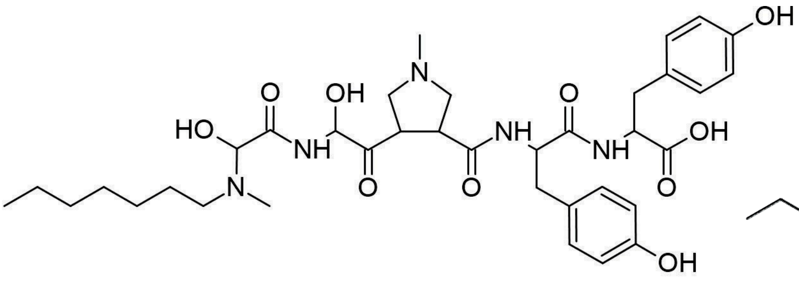

Microginin 712

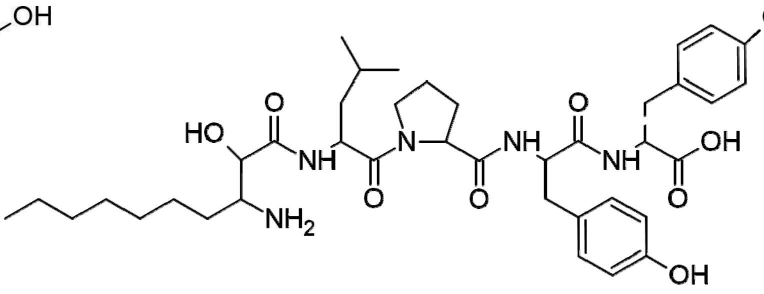

Microginin 754

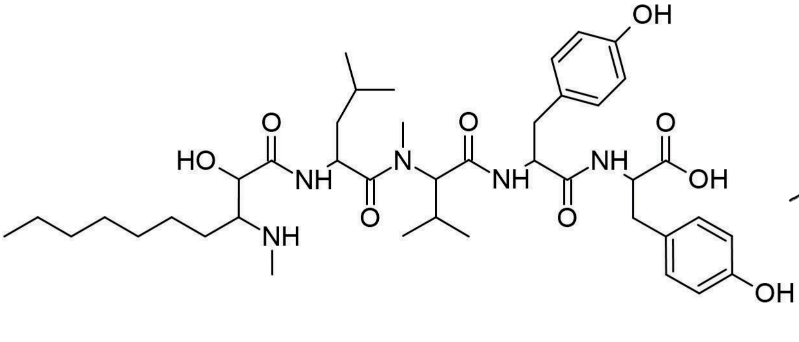

Microginin 784

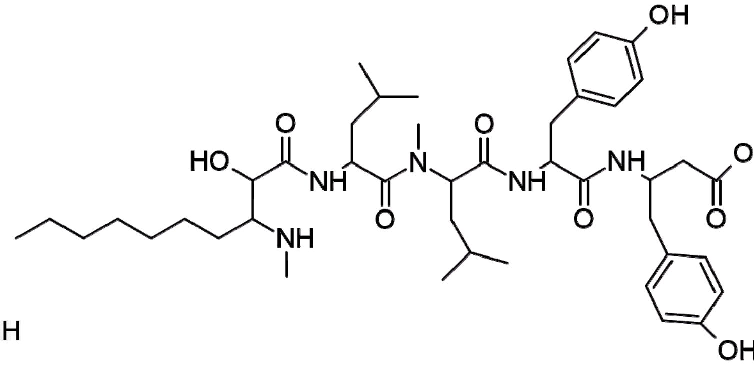

Microginin 798

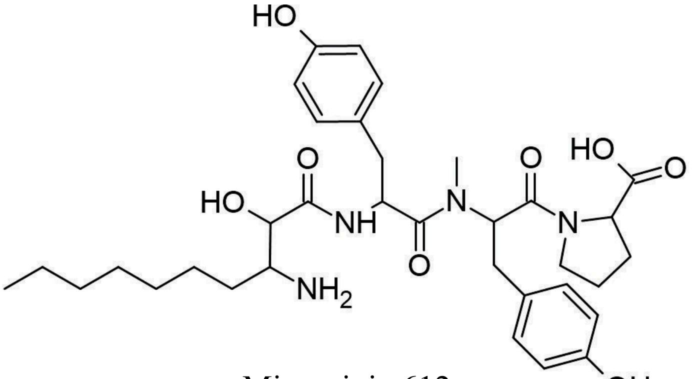

Microginin 612

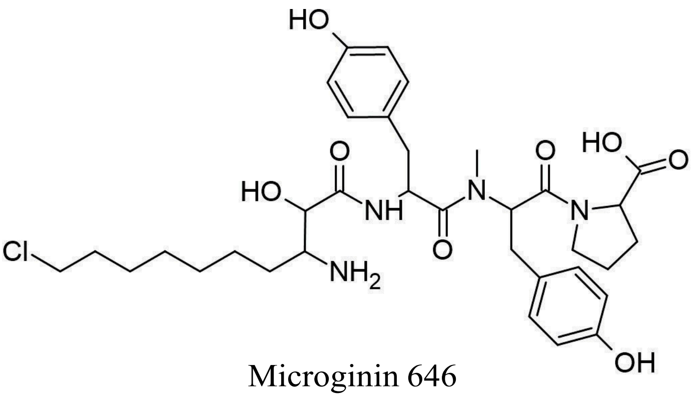

Microginin 646

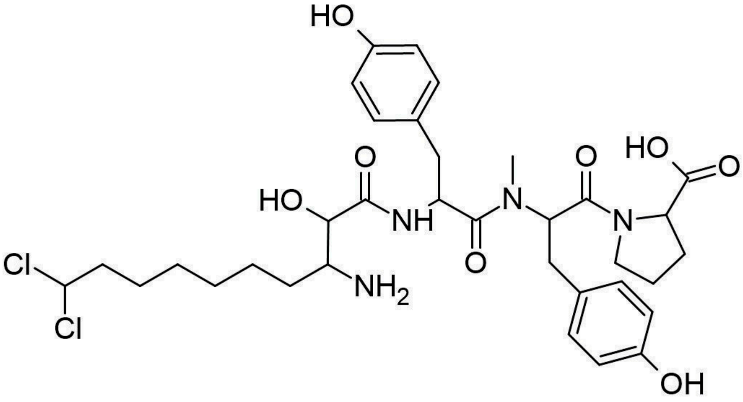

Microginin 680

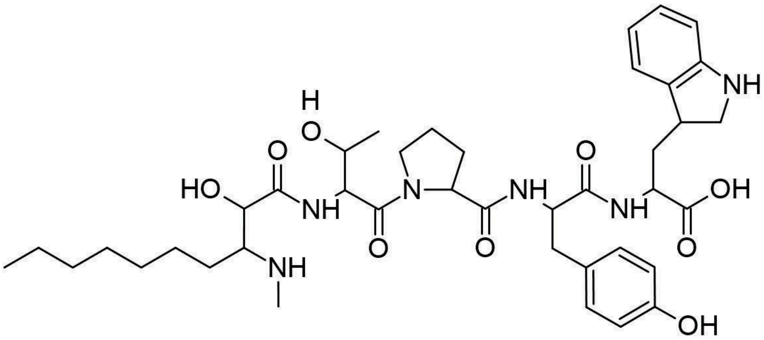

Microginin 764

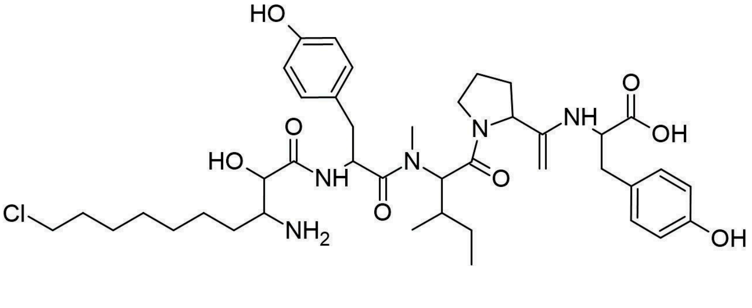

Microginin GH787

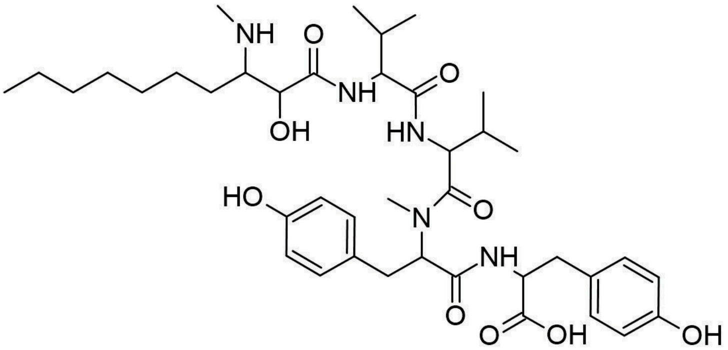

Microginin 755C

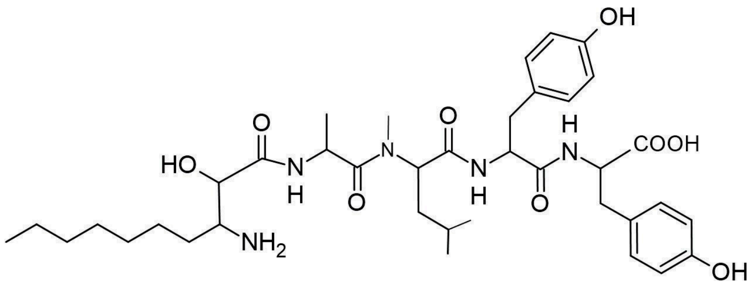

Microginin 727

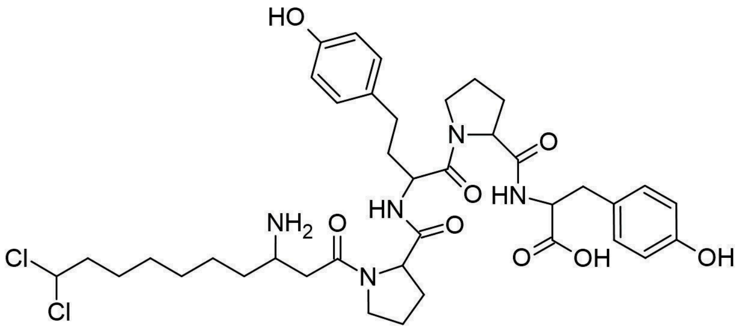

Microginin 789

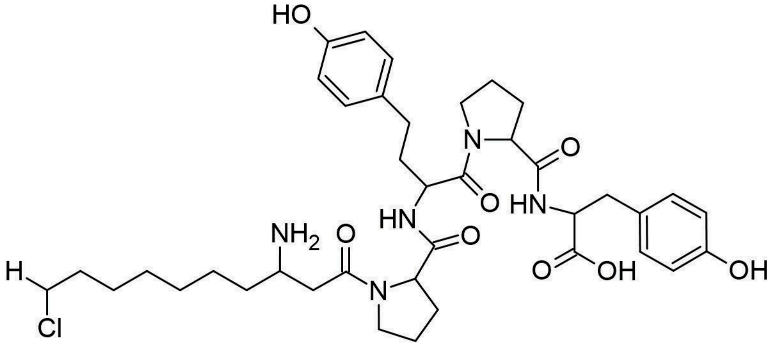

Microginin 755

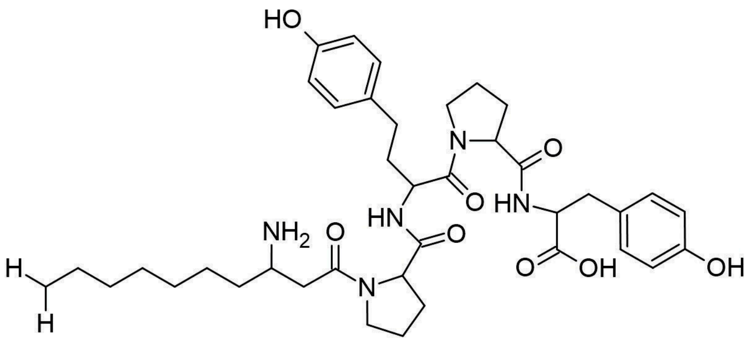

Microginin 721

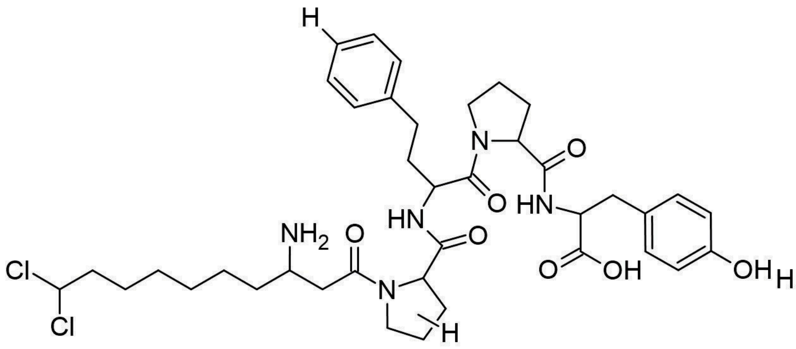

Microginin 773

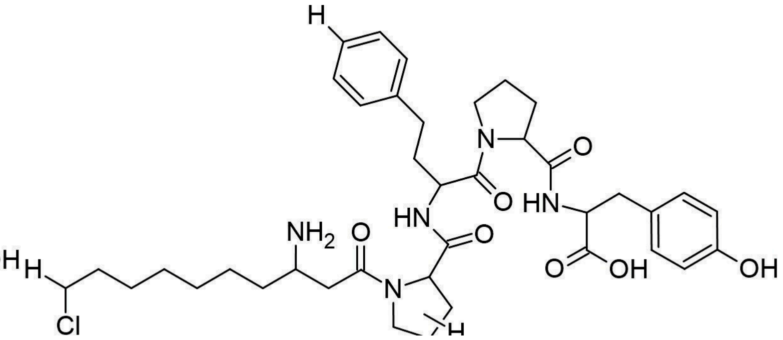

Microginin 739

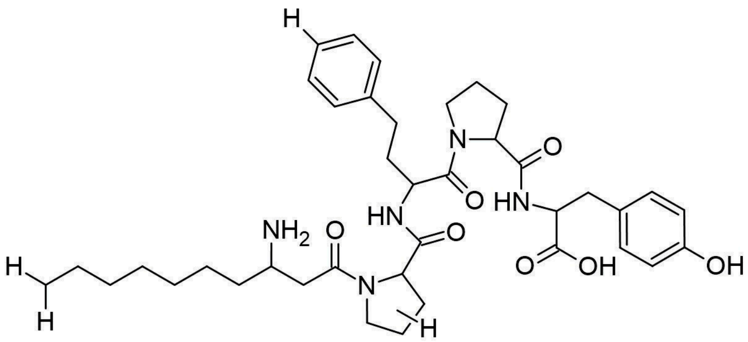

Microginin 705

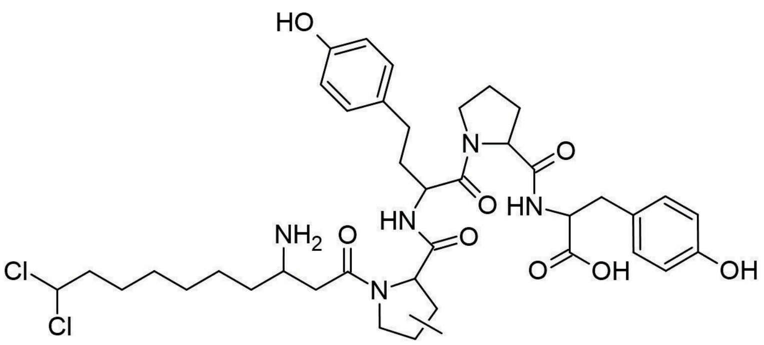

Microginin 803

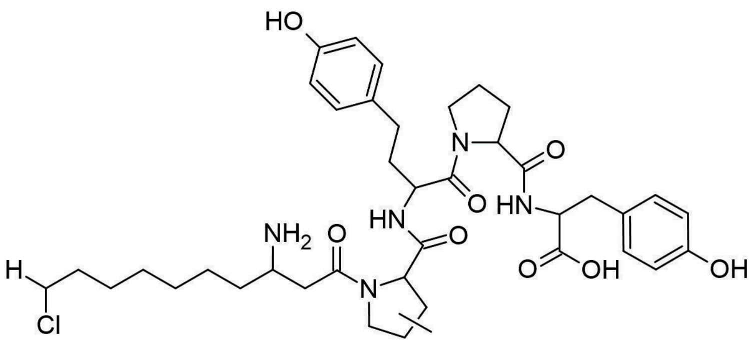

Microginin 769

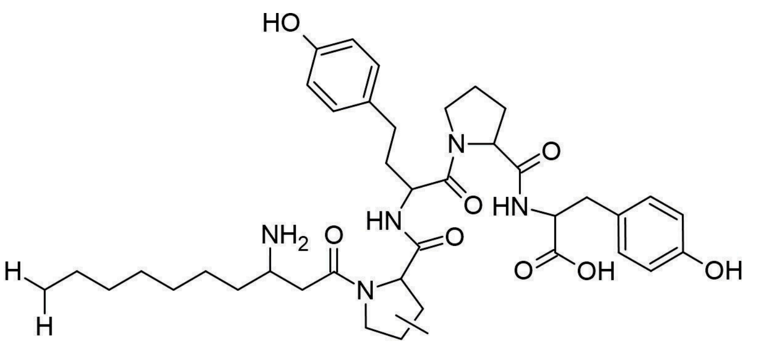

Microginin 735

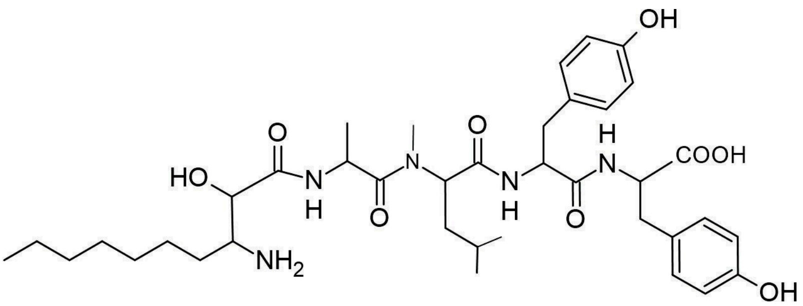

Microginin 727

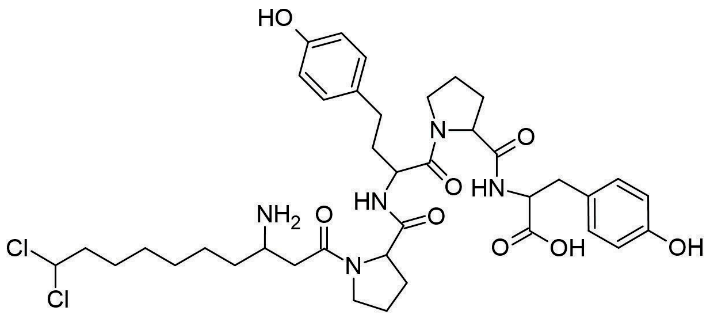

Microginin 789

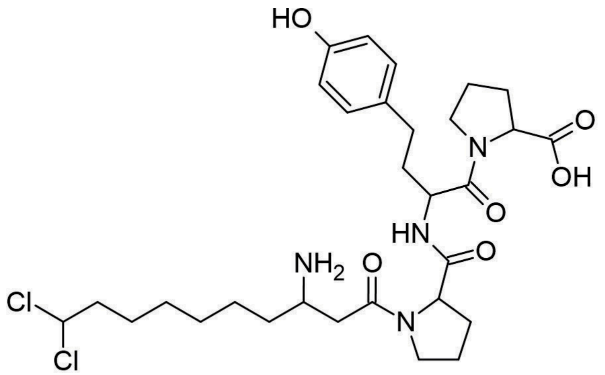

Microginin 626

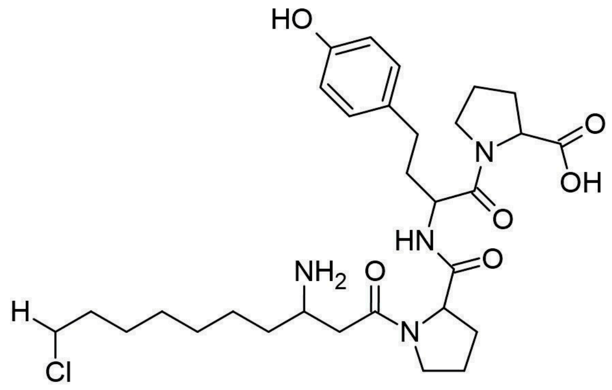

Microginin 592

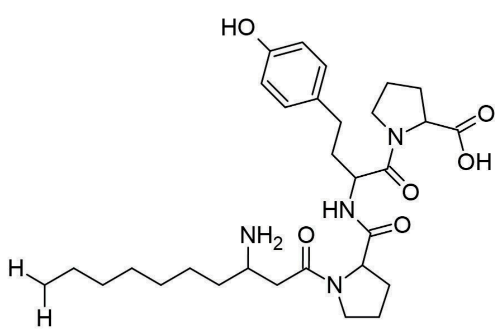

Microginin 558

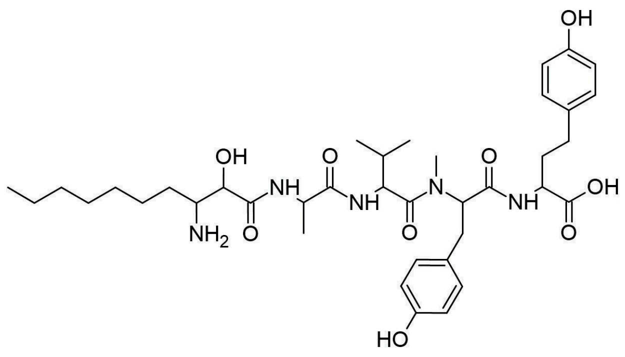

Cyanostatin A

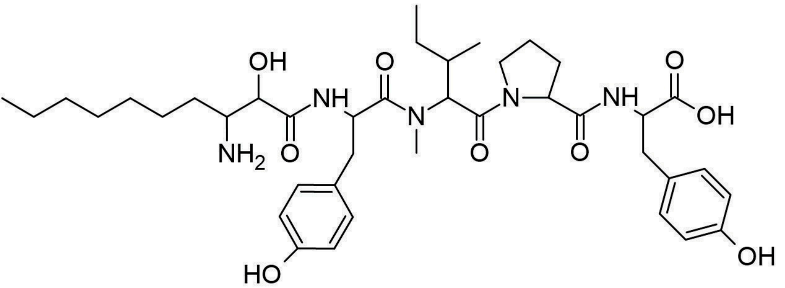

Cyanostatin B

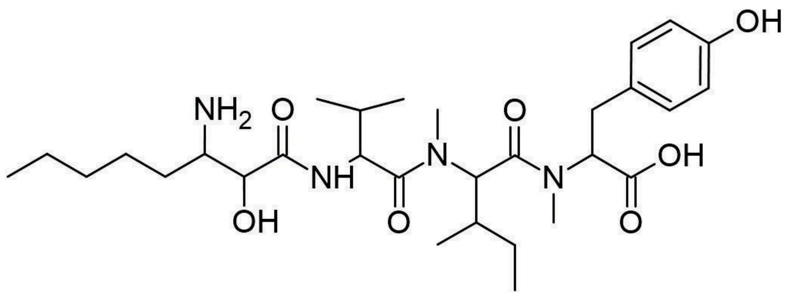

Nostoginin BN578

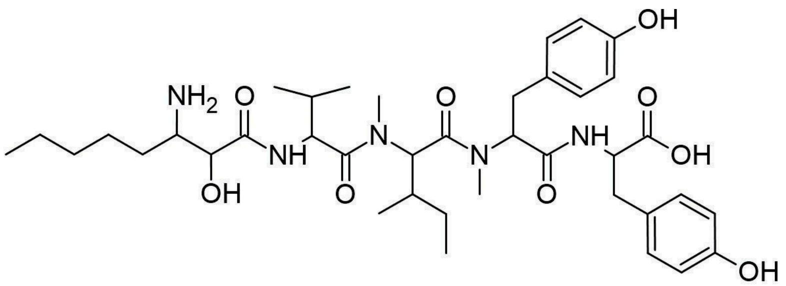

Nostoginin BN741

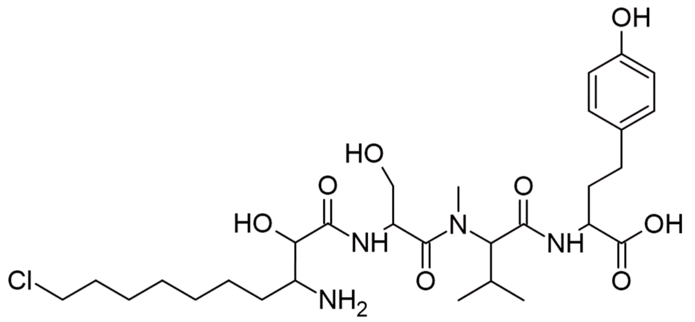

Oscillaginin A

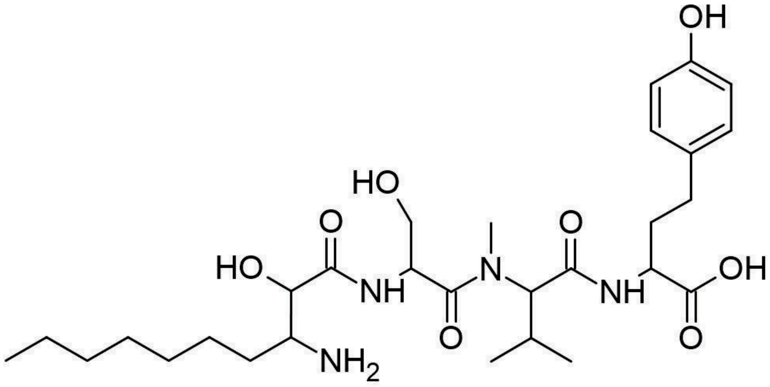

Oscillaginin B
