# Supplementary material for: Chemodiversity and Biotechnological Potential of Microginins
Source: Int J Mol Sci. 2025 Jun 25;26(13):6117. doi: 10.3390/ijms26136117 (PMC12250027; doi:10.3390/ijms26136117)
Supplement: Supplementary file 1 [file ijms-26-06117-s001.zip › Table S1 - Microginin ocurrence.pdf]

Table S1. Occurence and characterization of microginin in cyanobacteria.

N.	$[M+H]^+$(*m/z*)	Name	Sequence of amino acid						Species	Isolation source	Ref.
			1	2	3	4	5	6			
1	931.12	Microginin 51-B	MeAhda	Tyr	MeVal	MeTyr	Pro	Tyr	*M. aeruginosa* TAC-51	Lake Suwa (Japan)	[16]
2	921.94	Microginin 299-B	Cl_2Ahda	Val	MeVal	MeTyr	Pro	Tyr	*M. aeruginosa* (NIES-299)	Lake Kasumigaura (Japan)	[21]
3	917.10	Microginin 51-A	Ahda	Tyr	MeVal	MeTyr	Pro	Tyr	*M. aeruginosa* TAC-51	Lake Suwa (Japan)	[16]
4	887.50	Microginin 299-A	ClAhda	Val	MeVal	MeTyr	Pro	Tyr	*M. aeruginosa* (NIES-299)	Lake Kasumigaura (Japan)	[21]
5	853.05	Microginin 299-C	Ahda	Val	MeVal	MeTyr	Pro	Tyr	*M. aeruginosa* (NIES-299)	Lake Kasumigaura (Japan)	[17]
6	836.85	Microginin KR835	MeCl_2Ahda	Tyr	MeLeu	Pro	Tyr	-	*Microcystis* sp. IL-405	Kishon Reservoir (Israel)	[41]
7	816.43	Microginin KR815	MeClAhda	Tyr	MeLeu	Pro	MeTyr	-	*Microcystis* sp. IL-405	Kishon Reservoir (Israel)	[41]
8	810.38	Microginin GH787	ClAhda	Tyr	MeIle	Pro	Tyr	-	*M. aeruginosa* TAU IL-347	Lake Kinneret (Israel)	[39]
9	806.81	Microginin 99-B	Cl_2Ahda	Tyr	Leu	MeTyr	Pro	-	*M. aeruginosa* (NIES-99)	Lake Suwa (Japan)	[17]
10	804.35	Microginin 803	Cl_2Ada	Pro	Tyr	MePro	Tyr	-	*M. aeruginosa* LEGE 91341	Lake Braças (Portugal)	[8]
11	802.41	Microginin KR801	MeClAhda	Tyr	MeLeu	Pro	Tyr	-	*Microcystis* sp. IL-405	Kishon Reservoir (Israel)	[41]

12	798.60	Microginin 798	MeAhda	Leu	MeLeu	Tyr	Tyr	-	*Microcystis* sp.	Lake Balaton (Hungary)	[58]
13	792.20	Microginin 791	ClAhda	Thr	MeLeu	Tyr	Tyr	-	*Microcystis* sp.	Lake Zazari (Greece)	[10]
14	790.33	Microginin 789	Cl_2Ada	Pro	Tyr	Pro	Tyr	-	*M. aeruginosa* LEGE 91341	Lake Braças (Portugal)	[8]
15	788.38	Microginin KR787	ClAhda	Tyr	MeLeu	Pro	Tyr	-	*Microcystis* sp. IL-405	Kishon Reservoir (Israel)	[41]
16	784.57	Microginin 784	MeAhda	Leu	MeVal	Tyr	Tyr	-	*Microcystis* sp.	Lake Balaton (Hungary)	[58]
17	784.48	Microginin FR12	MeAhda	Val	MeLeu	MeTyr	Tyr	-	*M. aeruginosa* CPCC 299	Lake Champlain (USA)	[59]
18	780.45	Microginin KR781	MeAhda	Tyr	MeLeu	Pro	MeTyr	-	*Microcystis* sp. IL-405	Kishon Reservoir (Israel)	[41]
19	776.30	Microginin 776	Ahda	Ala	MeTyr	Phe	Tyr	-	*Microcystis* sp. TAU-MAC 1710	Lake Amvrakia (Greece)	[10]
20	774.34	Microginin 773	Cl_2Ada	Pro	Phe	Pro	Tyr	-	*M. aeruginosa* LEGE 91341	Lake Braças (Portugal)	[8]
21	772.81	Microginin 91-E	Cl_2Ahda	Ile	MeIle	Pro	Tyr	-	*M. aeruginosa* (NIES-478)	Lake Kasumigaura (Japan)	[16]
22	772.37	Microginin 99-A	ClAhda	Tyr	Leu	MeTyr	Pro	-	*M. aeruginosa* (NIES-99)	Lake Suwa (Japan)	[17]
23	772.20	Microginin 772	Ahda	Thr	MeLeu	Tyr	MeTyr	-	*M. aeruginosa*	Lake Kerkini (Greece)	[10]
24	770.50	Microginin 770	MeAhda	Val	Leu	Tyr	Tyr	-	*Microcystis* sp. LTPNA08/09	Salto Grande Reservoir (Brazil)	[33]
25	770.46	Microginin FR13	MeAhda	Val	MeIle	MeTyr	Tyr	-	*M. aeruginosa* CPCC 299	Lake Champlain (USA)	[59]

26	770.39	Microginin 769	ClAda	Pro	Tyr	MePro	Tyr	-	*M. aeruginosa* LEGE 91341	Lake Braças (Portugal)	[8]
27	769.96	Microginin 478	MeAhda	Val	MeVal	MeTyr	Tyr	-	*M. aeruginosa* (NIES-478)	Lake Kasumigaura (Japan)	[16]
28	767.97	Microginin 767	MeAhda	Tyr	MeLeu	Pro	Tyr	-	N.I.	Lake Auensee (Germany)	[35]
29	767.97	Microginin KR767	MeAhda	Tyr	MeLeu	Pro	Tyr	-	*Microcystis* sp. IL-405	Kishon Reservoir (Israel)	[41]
30	767.47	Microginin FR8	MeAhda	Pro	MeLeu	Tyr	Tyr	-	N.I.	N.I.	N.I.
31	766.10	Microginin 765	Cl_2Ahda	Ala	Pro	Tyr	Tyr	-	*Microcystis* sp.	Lake Kastoria (Greece)	[10]
32	765.42	Microginin 764	MeAhda	Thr	Pro	Tyr	Trp	-	*Microcystis* sp.	Lake Balaton (Hungary)	[58]
33	762.20	Microginin 761B	ClAhda	Thr	Pro	Tyr	Tyr	-	*Microcystis* sp.	Lake Kastoria (Greece)	[10]
34	762.0	Microginin 761A	ClAhda	Ala	MeLeu	Tyr	Tyr	-	*Microcystis* sp.	Lake Kastoria (Greece)	[10]
35	758.20	Microginin 757B	Cl_2Ahda	Pro	Val	MeLeu	Tyr	-	*M. aeruginosa*	Lake Zazari (Greece)	[10]
36	757.93	Microginin 757	Ahda	Thr	MeLeu	Tyr	Tyr	-	N.I.	Lake Auensee (Germany)	[35]
37	757.76	Microginin 299-D	Cl_2Ahda	Val	MeVal	MeTyr	Pro	-	*M. aeruginosa* (NIES-299)	Lake Kasumigaura (Japan)	[17]
38	756.45	Microginin 755C	MeAhda	Val	Val	MeIle	Tyr	Tyr	Cyanobacterial bloom	Lake Qaraoun (Lebanon)	[29]
39	756.40	Microginin 756B	MeAhda	Ala	MeLeu	MeTyr	Tyr	-	*Microcystis* sp.	Lake Pamvotida (Greece)	[10]

40	756.37	Microginin 755	ClAda	Pro	Tyr	Pro	Tyr	-	*M. aeruginosa* LEGE 91341	Lake Braças (Portugal)	[8]
41	755.93	Microginin 756	Ahda	Val	Leu	Tyr	Tyr	-	*Microcystis* sp. LTPNA08/09	Salto Grande Reservoir (Brazil)	[33]
42	755.47	Microginin SD-755	MeAhda	Val	MeIle	MeTyr	Tyr	-	N.I.	Samuel Reservoir (Brazil)	[61]
43	754.55	Microginin 754	Ahda	Leu	Pro	Tyr	Tyr	-	*Microcystis* sp.	Lake Balaton (Hungary)	[58]
44	754.43	Cyanostatin B	Ahda	Tyr	MeIle	Pro	Tyr	-	Cyanobacterial bloom	Loch Rescobie (Scotland)	[24]
45	750.89	Microginin FR9	Ahda	Thr	Pro	Tyr	Trp	-	N.I.	Lake Auensee (Germany)	[35]
46	748.92	Microginin FR10	Ahda	Val	Pro	Tyr	Trp	-	N.I.	N.I.	N.I.
47	748.32	Microginin 747B	ClAhda	Ala	Val	MeTyr	Tyr	-	*M. aeruginosa*	Lake Pamvotida (Greece)	[10]
48	748.27	Microginin 747A	ClAhda	Ser	Pro	Tyr	Tyr	-	*Microcystis* sp.	Lake Pamvotida (Greece)	[10]
49	744.41	Microginin FR7	Ahda	Ser	MeLeu	Tyr	Tyr	-	*M. aeruginosa* CPCC 299	Lake Champlain (USA)	[59]
50	744.40	Microginin 744	Ahda	Ser	MeLeu	Tyr	Tyr	-	*Microcystis* sp.	Lake Pamvotida (Greece)	[10]
51	742.40	Microginin FR4	MeAhda	Thr	Pro	Tyr	Tyr	-	N.I.	Lake Auensee (Germany)	[35]
52	742.30	Microginin 742A	MeAhda	Ala	MeLeu	Tyr	Tyr	-	*Microcystis* sp.	Lake Amvrakia (Greece)	[10]
53	742.30	Microginin 742B	MeAhda	Ser	Pro	Tyr	MeTyr	-	*Microcystis* sp.	Lake Kerkini (Greece)	[10]

54	742.0	Microginin 742C	Ahda	Ala	MeLeu	MeTyr	Tyr	-	*Nostoc oryzae* TAU-MAC 2710	Lake Pamvotida (Greece)	[10]
55	741.92	Nostoginin BN741	Ahoa	Val	MeIle	MeTyr	Tyr	-	*Nostoc* sp. TAU IL235	Jordan River (Israel)	[25]
56	741.88	Microginin FR2	MeAhda	Thr	Pro	Tyr	Tyr	-	*M. aeruginosa* NPCD-01	Samuel Reservoir (Brazil)	[61]
57	740.37	Microginin 739	ClAda	Pro	Phe	Pro	Tyr	-	*M. aeruginosa* LEGE 91341	Lake Braças (Portugal)	[8]
58	740.30	Microginin 740B	Ahda	Val	Pro	Tyr	MeTyr	-	*M. wesenbergii*	Lake Kastoria (Greece)	[10]
59	739.91	Microginin FR6	MeAhda	Val	Pro	Tyr	Tyr	-	N.I.	Lake Auensee (Germany)	[35]
60	738.36	Microginin 91-D	ClAhda	Ile	MeIle	Pro	Tyr	-	*M. aeruginosa* (NIES-478)	Lake Kasumigaura (Japan)	[16]
61	736.42	Microginin 735	Ada	Pro	Tyr	MePro	Tyr	-	*M. aeruginosa* LEGE 91341	Lake Braças (Portugal)	[8]
62	732.34	Microginin T1	ClAhda	Ala	Pro	Tyr	Tyr	-	*Microcystis* sp. *water bloom*	Lake Teganuma (Japan)	[23]
63	730.30	Microginin 730	Ahda	Ser	Val	MeTyr	Tyr	-	*Microcystis* sp.	Lake Kerkini (Greece)	[10]
64	728.42	Cyanostatin A	Ahda	Ala	Val	MeTyr	Tyr	-	Cyanobacterial bloom	Loch Rescobie (Scotland)	[24]
65	727.86	Microginin FR3	Ahda	Thr	Pro	Tyr	Tyr	-	N.I.	Lake Auensee (Germany)	[35]
66	727.0	Microginin FR1	Ahda	Ala	MeLeu	Tyr	Tyr	-	*Microcystis* sp. water bloom	Lake Waltershofen (Germany)	[43]
67	727.0	Microginin 727	Ahda	Ala	MeLeu	Tyr	Tyr	-	Cyanobacterial bloom	Lake Qaraoun (Lebanon)	[29]

68	726.41	Microginin FR5	Ahda	Val	Pro	Tyr	Tyr	-	N.I.	Lake Auensee (Germany)	[35]
69	726.35	Microginin 725	ClAhda	Ala	MeLeu	MeLeu	Tyr	-	*Microcystis* sp. TAU-MAC 1710	Lake Amvrakia (Greece)	[10]
70	722.42	Microginin 721	Ada	Pro	Tyr	Pro	Tyr	-	*M. aeruginosa* LEGE 91341	Lake Braças (Portugal)	[8]
71	718.30	Microginin 717	Cl_2Ahda	Ala	Val	Leu	Tyr	-	*M. aeruginosa*	Lake Kastoria (Greece)	[10]
72	716.30	Microginin 715	ClAhda	Val	Pro	Tyr	Tyr	-	*Microcystis* sp.	Lake Kerkini (Greece)	[10]
73	714.40	Microginin 1	Ahda	Ala	Val	MeTyr	Tyr	-	*M. aeruginosa* (NIES-100)	Lake Suwa (Japan)	[7]
74	714.20	Microginin 714B	Ahda	Ser	Pro	Tyr	Tyr	-	*Microcystis* sp.	Lake Kerkini (Greece)	[10]
75	712.52	Microginin 712	Ahda	Ala	MePro	Tyr	Tyr	-	*Microcystis* sp.	Lake Balaton (Hungary)	[58]
76	711.46	Microginin 711	Ahda	Val	MeLeu	Tyr	Tyr	-	N.I.	N.I.	[10]
77	706.42	Microginin 705	Ada	Pro	Phe	Pro	Tyr	-	*M. aeruginosa* LEGE 91341	Lake Braças (Portugal)	[8]
78	704.88	Microginin 704	Ahda	MeMet(O)	Tyr	TyrOMe	-	-	*M. aeruginosa* UTEXLB2385	Lake Little Rideau (Canada)	[18]
79	703.92	Microginin 91-C	Ahda	Ile	MeIle	Pro	Tyr	-	*M. aeruginosa* (NIES-478)	Lake Kasumigaura (Japan)	[16]
80	698.38	Microginin T2	Ahda	Ala	Pro	Tyr	Tyr	-	*Microcystis* sp. water bloom	Lake Teganuma (Japan)	[23]
81	690.85	Microginin 690	Ahda	Tyr	MeMet	Tyr	-	-	*M. aeruginosa* UTEXLB2385	Lake Little Rideau (Canada)	[18]

82	683.64	Microginin 683	ClAhda	Ala	Val	Leu	Tyr	-	*M. aeruginosa*	Lake Kastoria (Greece)	[10]
83	681.24	Microginin 680	Cl_2Ahda	Tyr	MeTyr	Pro	-	-	*M. aeruginosa* UTEX LB2386	Lake Little Rideau (Canada)	[40]
84	674.85	Microginin 674	Ahda	Tyr	MeMet	Tyr	-	-	*M. aeruginosa* UTEXLB2385	Lake Little Rideau (Canada)	[18]
85	650.0	Microginin 650	Ahda	Ala	Val	Leu	Tyr	-	N.I.	N.I.	[10]
86	647.28	Microginin 646	ClAhda	Tyr	MeTyr	Pro	-	-	*M. aeruginosa* UTEX LB2386	Lake Little Rideau (Canada)	[40]
87	638.34	Microginin KR638	MeClAhda	Tyr	MeLeu	Pro	-	-	*Microcystis* sp. IL-405	Kishon Reservoir (Israel)	[41]
88	627.27	Microginin 626	Cl_2Ada	Pro	Tyr	Pro	-	-	*M. aeruginosa* LEGE 91341	Lake Braças (Portugal)	[8]
89	621.40	Microginin 621B	MeAhda	Leu	MeLeu	Tyr	-	-	*Microcystis* sp.	Lake Amvrakia (Greece)	[10]
90	621.30	Microginin 621A	MeAhda	Val	MeLeu	MeTyr	-	-	*M. aeruginosa*	Lake Mikri Prespa (Greece)	[10]
91	615.31	Oscillaginin A	ClAhda	Ser	MeVal	Tyr	-	-	*O. agardhii* NIES-610	Lake Gjersjøen (Norway)	[24]
92	613.32	Microginin 612	Ahda	Tyr	MeTyr	Pro	-	-	*M. aeruginosa* UTEX LB2386	Lake Little Rideau (Canada)	[40]
93	609.63	Microginin 91-B	Cl_2Ahda	Ile	MeIle	Pro	-	-	*M. aeruginosa* (NIES-478)	Lake Kasumigaura (Japan)	[16]
94	607.46	Microginin 607A	MeAhda	Val	Leu	MeTyr	-	-	*Microcystis* sp.	Lake Kastorias (Greece)	[10]
95	607.25	Microginin 607B	Ahda	Val	MeLeu	MeTyr	-	-	*M. aeruginosa*	Lake Mikri Prespa (Greece)	[10]

96	605.20	Microginin 605	ClAhda	-	Pro	Tyr	-	-	N.I.	Lake Kerkini (Greece)	[10]
97	604.79	Microginin KR604	MeAhda	Tyr	MeLeu	Pro	-	-	*Microcystis* sp. IL-405	Kishon Reservoir (Israel)	[41]
98	598.28	Microginin 598	ClAhda	Thr	Pro	Tyr	-	-	*Microcystis* sp.	Lake Pamvotida (Greece)	[10]
99	595.50	Microginin 595	Ahda	Thr	MePro	OHTyr	-	-	*Microcystis* sp.	Lake Balaton (Hungary)	[58]
100	593.31	Microginin 592	ClAda	Pro	Tyr	Pro	-	-	*M. aeruginosa* LEGE 91341	Lake Braças (Portugal)	[8]
101	591.20	Microginin 591A	MeAhda	-	MeLeu	Tyr	-	-	*Microcystis* sp.	Lake Amvrakia (Greece)	[10]
102	591.20	Microginin 591B	Ahda	Tyr	MeLeu	Pro	-	-	*Microcystis* sp.	Lake Pamvotida (Greece)	[10]
103	585.30	Microginin AL584	ClAhda	Ala	MeVal	MeTyr	-	-	*Microcystis* sp. TAU-IL306	Water reservoir Kibbutz (Israel)	[15]
104	581.50	Microginin 581B	Ahda	Thr	Pro	OHTyr	-	-	*Microcystis* sp.	Lake Balaton (Hungary)	[58]
105	581.38	Oscillaginin B	Ahda	Ser	MeVal	Tyr	-	-	*O. agardhii* NIES-610	Lake Gjersjøen (Norway)	[24]
106	581.20	Microginin 581	Ahda	Ser	MeLeu	Tyr	-	-	*Microcystis* sp.	Lake Amvrakia (Greece)	[10]
107	579.20	Microginin 579B	MeAhda	Thr	Pro	Tyr	-	-	*Microcystis* sp.	Lake Balaton (Hungary)	[58]
108	579.20	Microginin 579C	Ahda	Ala	MeTyr	MeLeu	-	-	*Microcystis* sp. TAU-MAC 0710	Lake Amvrakia (Greece)	[10]
109	579.0	Nostoginin BN578	Ahoa	Val	MeIle	MeTyr	-	-	*Nostoc* sp. TAU IL235	Jordan River (Israel)	[25]
110	578.90	Microginin 579A	MeAhda	Ala	MeLeu	Tyr	-	-	*Microcystis* sp.	Lake Amvrakia	[10]

111	577.20	Microginin 576	ClAhda	Ile	MeLeu	Val	-	-	*Microcystis* sp. TAU-MAC 0710	Lake Amvrakia (Greece)	[10]
112	575.19	Microginin 91-A	ClAhda	Ile	MeIle	Pro	-	-	*M. aeruginosa* (NIES-478)	Lake Kasumigaura (Japan)	[16]
113	568.26	Microginin 568	ClAhda	Ala	Pro	Tyr	-	-	N.I.	Lake Kerkini (Greece)	[10]
114	565.50	Microginin 565B	Ahda	Thr	Pro	Tyr	-	-	*Microcystis* sp.	Lake Balaton (Hungary)	[58]
115	565.37	Microginin 565A	Ahda	Ala	MeLeu	Tyr	-	-	*A. fertilissima* CCC597	N.I.	[60]
116	559.35	Microginin 558	Ada	Pro	Tyr	Pro	-	-	*M. aeruginosa* LEGE 91341	Lake Braças (Portugal)	[8]
117	551.50	Microginin 551	Ahda	Ser	Pro	Tyr	-	-	N.I.	Lake Kerkini (Greece)	[10]
118	549.50	Microginin 549	Ahda	Ala	MePro	Tyr	-	-	*Microcystis* sp.	Lake Balaton (Hungary)	[58]
119	535.50	Microginin 535	Ahda	Ala	Pro	Tyr	-	-	N.I.	Lake Amvrakia (Greece)	[10]
120	527.68	Microginin 527	Ahda	MeMet(O)	Tyr	-	-	-	*M. aeruginosa* UTEXLB2385	Lake Little Rideau (Canada)	[18]
121	511.68	Microginin 511	Ahda	MeMet	Tyr	-	-	-	*M. aeruginosa* UTEXLB2385	Lake Little Rideau (Canada)	[18]
